# Chimeric Proton-Pumping Rhodopsins Containing the Cytoplasmic Loop of Bovine Rhodopsin

**DOI:** 10.1371/journal.pone.0091323

**Published:** 2014-03-12

**Authors:** Kengo Sasaki, Takahiro Yamashita, Kazuho Yoshida, Keiichi Inoue, Yoshinori Shichida, Hideki Kandori

**Affiliations:** 1 Department of Frontier Materials, Nagoya Institute of Technology, Showa-ku, Nagoya, Japan; 2 Department of Biophysics, Graduate School of Science, Kyoto University, Kyoto, Japan; 3 PRESTO, Japan Science and Technology Agency, Honcho Kawaguchi, Saitama, Japan; University of Oldenburg, Germany

## Abstract

G-protein-coupled receptors (GPCRs) transmit stimuli to intracellular signaling systems. Rhodopsin (Rh), which is a prototypical GPCR, possesses an 11-*cis* retinal. Photoisomerization of 11-*cis* to all-*trans* leads to structural changes in the protein of cytoplasmic loops, activating G-protein. Microbial rhodopsins are similar heptahelical membrane proteins that function as bacterial sensors, light-driven ion-pumps, or light-gated channels. They possess an all-*trans* retinal, and photoisomerization to 13-*cis* triggers structural changes in protein. Despite these similarities, there is no sequence homology between visual and microbial rhodopsins, and microbial rhodopsins do not activate G-proteins. In this study, new chimeric proton-pumping rhodopsins, proteorhodopsin (PR) and *Gloeobacter* rhodopsin (GR) were designed by replacing cytoplasmic loops with bovine Rh loops. Although G-protein was not activated by the PR chimeras, all 12 GR chimeras activated G-protein. The GR chimera containing the second cytoplasmic loop of bovine Rh did not activate G-protein. However, the chimera with a second and third double-loop further enhanced G-protein activation. Introduction of an E132Q mutation slowed the photocycle 30-fold and enhanced activation. The highest catalytic activity of the GR chimera was still 3,200 times lower than bovine Rh but only 64 times lower than amphioxus Go-rhodopsin. This GR chimera showed a strong absorption change of the amide-I band on a light-minus-dark difference FTIR spectrum which could represent a larger helical opening, important for G-protein activation. The light-dependent catalytic activity of this GR chimera makes it a potential optogenetic tool for enzymatic activation by light.

## Introduction

Rhodopsin (Rh) is one of the G protein-coupled receptors (GPCRs) that has diverged into a photoreceptive protein in retinal visual cells [1]–[6]. It is a membrane protein consisting of a single polypeptide opsin and a light-absorbing chromophore 11-*cis*-retinal. Opsin contains seven transmembrane α-helices, which form a structural motif typical of GPCRs. An 11 *cis* retinal is bound to Lys-296 in the transmembrane helix 7 through a protonated Schiff base linkage. Absorption of a photon by the chromophore causes isomerization to the all-*trans* form, followed by conformational changes to the protein [1], [3]. Several intermediate states in the bleaching process have been identified as photorhodopsin, bathorhodopsin (Batho), lumirhodopsin (Lumi), metarhodopsin-I (Meta-I), and metarhodopsin-II (Meta-II). Meta-II catalyzes the GDP-GTP exchange reaction in the transduction of trimeric G protein (Gt) [1], [7], [8].

Some archaea and bacteria possess retinal binding proteins, which are microbial rhodopsins that contain an all-*trans* retinal as a chromophore. Among them, the most studied is bacteriorhodopsin (BR), which is found in *Halobacterium salinarum* [3], [9]–[11]. This archaea contains four retinal-bonding proteins: BR, halorhodopsin (HR), sensory rhodopsin I (SRI) and sensory rhodopsin II (SRII; also called phoborhodopsin) [12]. BR and HR are light-driven ion pumps, acting as an outward proton- and an inward chloride-pump, respectively [9], [12]–[14]. SRI and SRII, which are photoreceptors of this halophilic archaea, act in attractant and repellent responses, respectively during phototaxis [15], [16].

There are no sequence homologies between visual and microbial rhodopsins, though both possess similar chromophore (retinal) and protein (7-transmembrane helices) structures. It is generally believed that both evolved independently, although the most recent study suggests possibility convergent evolution between both types of rhodopsins [17].) In fact, visual rhodopsins do not transport ions, while microbial rhodopsins do not activate G-proteins. However, Geiser et al. reported that BR chimeras containing the third cytoplasmic loop of bovine Rh are able to activate G-protein [18]. Although the activation level was reported to be similar between native bovine Rh and the chimera (BR/Rh223-253), we recently quantified the activation level to be 0.003% of bovine Rh [19]. In addition, we reported that chimeras of *Natronomonas pharaonis* SRII containing third loops of bovine Rh are able to activate G-protein [19]. This observation suggests that a common structural feature for light-induced activation among BR, SRII and bovine Rh exists in which the helix opening motion on the cytoplasmic side probably exposes the third loop to possible binding with G-protein.

These chimeras have the potential to be tools for optogenetics [20], [21]. In fact, Arian et al. developed a versatile family of genetically encoded optical tools (‘optoXRs’), in which a bovine Rh chimera containing the cytoplasmic loop of other GPCRs was used to respond to light [22]. Even though Rh may be a promising optogenetic tool, it has no photocycle, but bleaches upon light absorption [6]. In addition, 11-*cis* retinal, the chromophore molecule of Rh, is not generally abundant in animal cells. This limits the practical application of ‘optoXRs’. In contrast, our chimera containing an all-*trans* retinal exhibited a photocycle without any bleaching and was being repeatedly applicable [19]. Endogenous all-*trans* retinal is sufficient for optogenetics in animal cells [14], [23]. Thus, our chimeras can be tested for light-induced enzymatic activation in optogenetics. The greatest problem of these chimeras is likely their low activation level (0.003% of bovine Rh) [19].

In this study, chimeric proton-pumping rhodopsins were designed using Proteorhodopsin (PR) from marine γ-proteobacteria [24] and *Gloeobacter* rhodopsin (GR) from thylakoidless cyanobacteria to make new microbial rhodopsin chimeras with a higher G-protein activating function [25], [26].

## Experimental Procedures

### Sample Preparation

**Figure 1:**
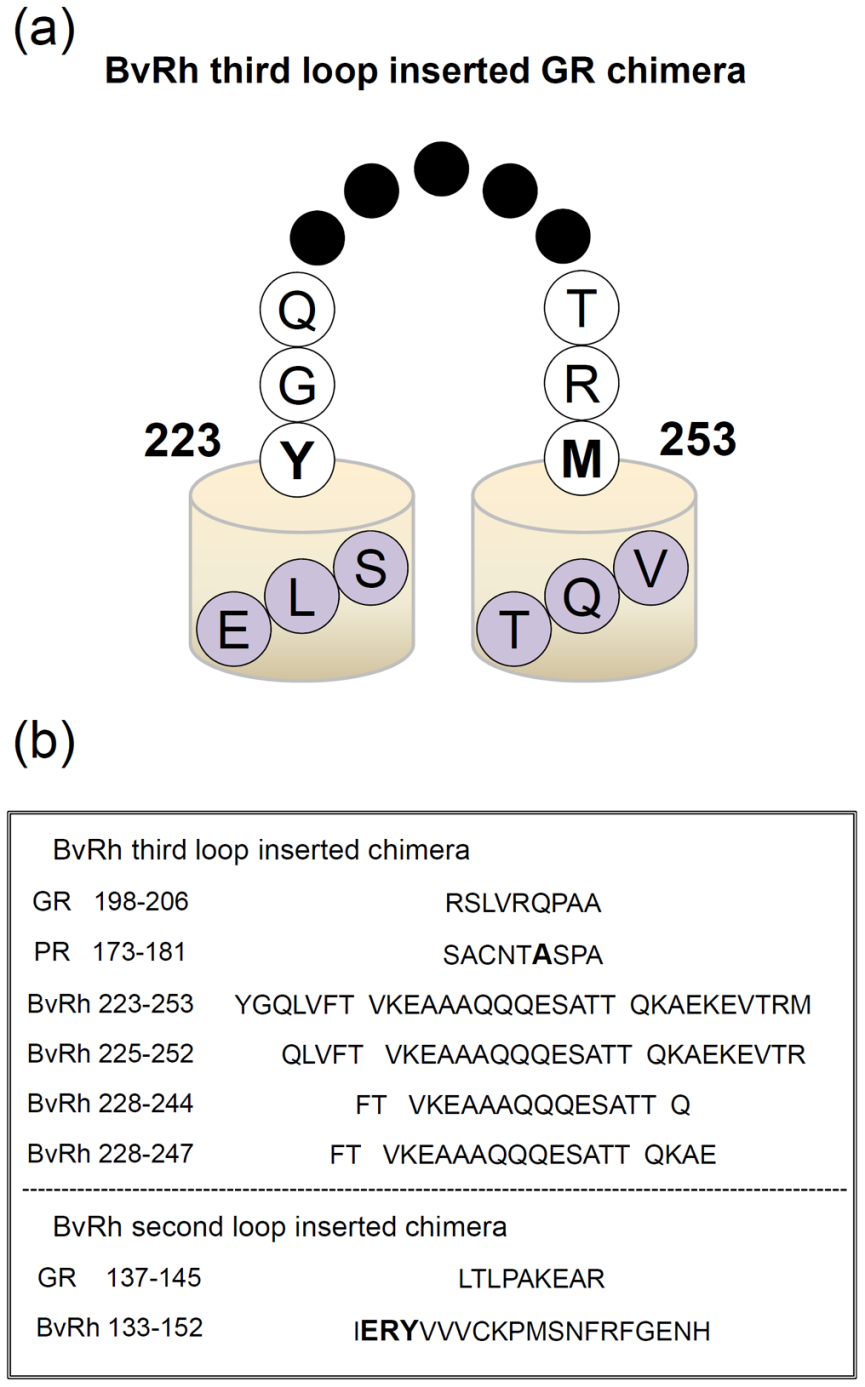
Design of chimeric proteins from PR and GR. (a) Schematic drawing of the secondary structure of the chimera. The GR sequence of the E-helix (…ELS) is connected to the third loop of bovine Rh, which further continues to the F-helix (…VQT) of GR. (b) We removed the sequence of RSLVRQPAA in the E–F loop from GR (SACNTASPA from PR), and inserted constructs of the third loop of bovine Rh. In the second loop, we removed the sequence of LTLPAKEAR in the C–D loop from GR. The A178 position of PR and the ERY residue of bovine Rh are emphasized in bold.

Chimera constructs were designed based on the wild-type (WT) PR (triple cysteine mutant, TCM) and GR (Figure 1) (Sequential alignment among previously studied rhodopsins (BR and SRII), PR and GR is shown in Figure S1). The DNA template of the bovine Rh loop was exchanged by the following three-step PCR. At first, three PCR products were constructed and purified: the front side of PR before the exchange region and to which a top 15 mer Rho loop was added by a primer to the end (fPR), the last side of PR remained the same (lPR), and the loop region of bovine Rh amplified by a primer with an additional PR 15-mer near the exchange region (Rh loop). The products of the first PCR were used to amplify a second round PCR product. Then, fPR was extended to the loop region by PCR with an Rh loop and the same lPR. Finally, the final full-length chimera fragment amplified the former two products which were cloned into pET21c (pKJ900 in GR) vector by inserting with *Nde*I/*Xho*I (*Xba*I/*Not*I) digestion. After ligation, the plasmids were transformed into *Escherichia coli* JM109 strain. All chimeras were confirmed by DNA sequencing (Hokkaido System Science, Japan). The WT and chimeric proteins of PR possessing a six histidine tag at the C-terminus were expressed in *E. coli*, solubilized with 1% *n*-dodecyl-β-D-maltoside (DDM), and purified by Co^2+^-column chromatography as described previously [27]. Absorption spectra of solubilized proteins (300 mM NaCl, 300 mM imidazole, 50 mM Tris-HCl, pH 7.0 and 0.1% DDM) were measured at 20°C using a UV-visible spectrophotometer (UV-2400PC, Shimadzu).

Wild-type bovine rhodopsin was expressed in HEK293S cells and was purified by using the monoclonal antibody rho1D4 against the C-terminal sequence of bovine rhodopsin [28]–[30]. A G-protein transducin was purified from bovine rod outer segments (ROS) according to the method previously described [31].

### G-Protein Activation Assays

A radionucleotide filter-binding assay, which measures the light-dependent exchange of GDP/GTPγS by transducin, was carried out as described previously [32]. All procedures were carried out at 20°C. The assay mixture consisted of 50 mM HEPES (pH 7.0), 140 mM NaCl, 5 mM MgCl_2_, 1 mM DTT, 0.05% DDM, 1 μM [^35^S]GTPγS and 2 μM GDP. The mixture of rhodopsin (final concentration; 2.1 μM PR, 1.9 μM GR, and 5 nM bovine Rh) and transducin (final concentration; 600 nM) was constantly irradiated with white light or was kept in the dark. After incubation for a selected period of time, an aliquot (20 μl) was removed from the sample and placed into 200 μl of stop solution (20 mM Tris/Cl (pH 7.4), 100 mM NaCl, 25 mM MgCl_2_, 1 μM GTPγS and 2 μM GDP), which it was immediately filtered through a nitrocellulose membrane to trap [^35^S]GTPγS bound to transducin. The amount of bound [^35^S]GTPγS was quantified by assaying the membrane with a liquid scintillation counter (Tri-Carb 2910 TR, Perkin Elmer).

### Pulsed Laser Flash Photolysis

Photocycles of WT and chimeric GR proteins were measured by using a flash photolysis apparatus as described previously [33], [34]. Purified GR sample (chimera and WT) was resuspended in buffer (300 mM NaCl, 300 mM imidazole, 50 mM Tris-HCl, pH 7.0 and 0.1% DDM). Flash-induced absorption changes were acquired with 40 points of logarithmic acquaintance and 20 milli-second intervals for GR and a long photocycle having GR/Rh225-252 + E132Q, respectively, by using a commercial flash photolysis system (Hamamatsu Photonics K.K., Japan), which consists of a CCD linear detector (Photonic Multichannel Spectral Analyzer PMA-11 C8808-01), a CW xenon lamp (L8004) as a light source and a sample room (Flash photolysis optics C9125). Each sample was excited with 532 nm nanosecond laser pulses from a Nd^3+^-YAG laser apparatus (INDI40, Spectra-Physics). The energy of one laser pulse was 0.13 mJ. The repetition rate of the laser was adjusted from 0.1–1 Hz to be sufficiently slower than the turnover rate of the photocycle in order to avoid unnecessary photo-absorption of the photoreaction intermediate. To improve the signal-to-noise ratio, 50 photoreactions were averaged for each sample solution of PR and GR. The absorbance was adjusted to 0.5 at the *λ*_max_ and the temperature of each sample was maintained at 25°C.

### Proton-Pump Measurement

The proton transport efficiency of GR WT and chimeras was measured by monitoring pH changes in the *E. coli* suspension in water containing 50 mM MgCl_2_ and 150 mM NaCl with a glass electrode [34], [35]. The *E. coli* cells were cultured by 50 ml 2×YT medium and the turbidity was OD ∼0.7 at 660 nm. The cells were washed for three times by the solvent for the measurement and 6 times concentrated. The initial pH was adjusted to 6.6–7.2 by the addition of small amount of HCl or NaOH. The samples were illuminated with a 1 kW tungsten-halogen projector lamp (Master HILUX-HR, Rikagaku, Japan) at >500 nm through a glass filter (AGC Techno Glass Y-52, Japan), and changes in pH value were monitored with an F-55 pH meter (Horiba, Japan).

### Light-Induced Difference FTIR Spectroscopy

Light-induced difference FTIR spectroscopy of GR, PR and their chimeras was performed as described previously [26], [36], [37]. Each protein was reconstituted into L-α-phosphatidylcholine liposomes by removing the detergent with Bio-beads in which the molar ratio of the added lipid to protein was 30∶1. The samples in PC liposomes were washed twice with a pH 7.0 buffer (2 mM phosphate, 5 mM NaCl). A 40-μl aliquot of the sample was deposited on a BaF_2_ window 18 mm in diameter and vacuum-dried in a glass vessel attached to an aspirator. The film sample was hydrated with 1 μL of H_2_O before measurements. Then, the sample was placed in a cryostat (Oxford DN-1704, UK) mounted in the FTIR spectrometer (Bio-Rad FTS-7000, USA). The cryostat was equipped with a temperature controller (Oxford ITC-4, UK) that regulated temperature with 0.1 K precision.

The sample was illuminated with 520±5 nm light at 250 K for 30 seconds, which converted each protein into the red-shifted O intermediate. The difference spectra after minus before illumination correspond to the O minus GR (GR chimeras) spectra, which were obtained with 2 cm^−1^ resolution. We averaged 1–8 independent measurements with 128 scans.

## Results

### Absorption Properties of the PR/Rh and GR/Rh Chimeras

**Figure 2:**
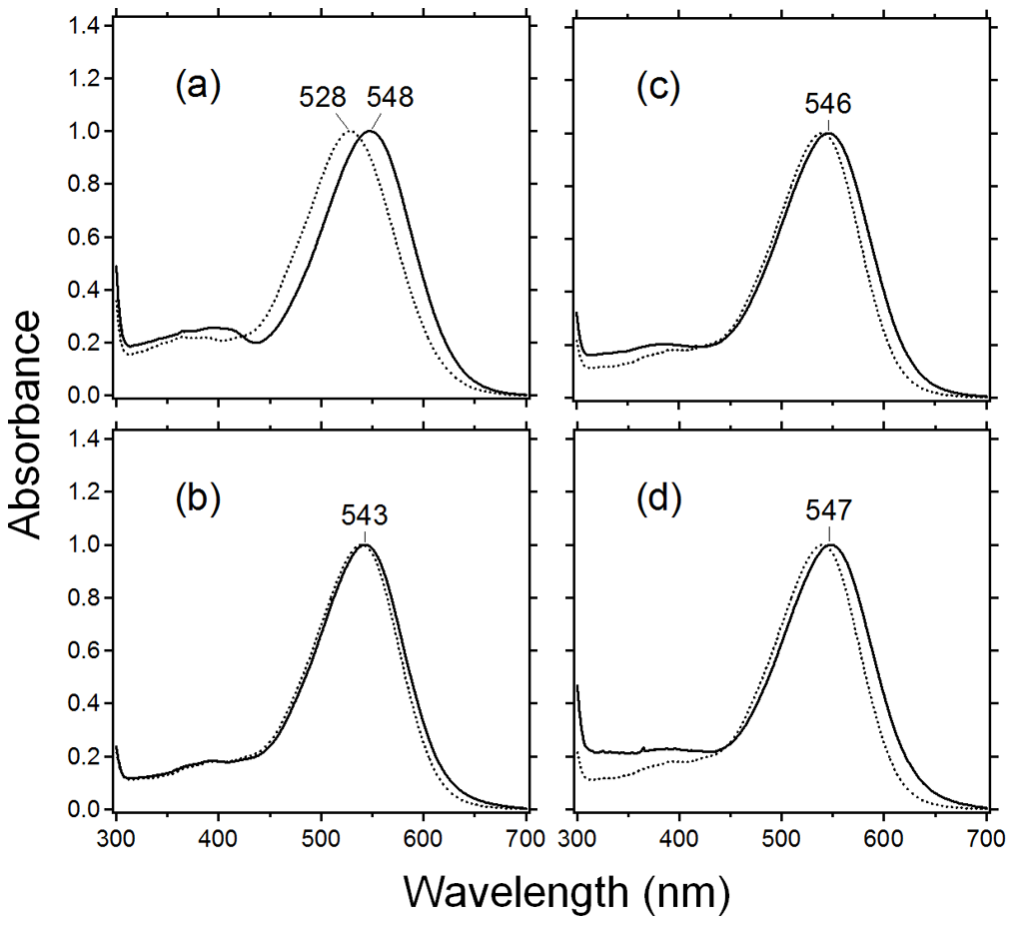
Absorption spectra of chimeras. (a) Absorption spectra of WT PR (dotted line) and PR/Rh225-252 (solid line). (b) – (d) Absorption spectra of WT GR (dotted line) and GR/Rh225-252 (solid line in (b)), GR/Rh133-152 (solid line in (c)), GR/Rh133-152 + 228244 (solid line in (d)). All samples were solubilized in a 0.1% DDM solution.

In the present study, we expressed the PR/Rh and GR/Rh chimera in *E. coli*, followed by solubilization with DDM and purification through a Co^2+^-NTA column. The schematic structure of the third loop of bovine Rh inserted into chimeras is shown in Figure 1a and the amino-acid sequences of the cytoplasmic loops of GR, PR and bovine Rh used for the construction of the chimeras are shown in Figure 1b. In the PR/Rh225-252 chimera, a 20-nm red shift (WT PR; 528 nm, PR/Rh chimera; 548 nm) was observed (Figure 2a). In fact, all other PR/Rh chimeras exhibited a 20-nm red-shifted *λ*_max_ (Figure S2). Although the loop region is distant from the retinal moiety, this observation is consistent with a previous observation in which an A178X mutation at the third cytoplasmic loop of PR causes a spectral red-shift [38], [39]. These studies revealed that the E–F loop region contains a unique structure in PR, disruption of which causes large-scale rearrangement of α-helices, and that A178 contributes to the blue-shifted absorption and lowering of the counterion pKa. Figure S3 shows that the pKa of the Schiff base counterion (D97) increased for all PR chimeras, which is also consistent with the A178X mutants [38], [39]. A mutation at the third cytoplasmic loop caused structural alteration at the Schiff base region and replacement of the third cytoplasmic loop by a bovine Rh loop caused the same effect.

In the GR/Rh225-252 chimera (the third loop chimera), a 3-nm red shift (WT GR; 540 nm, GR/Rh chimera; 543 nm) was observed (Figure 2b; the spectra of all GR/Rh chimeras constructed in this study are shown in Figure S4). This observation suggests the presence of a similar mechanism of spectral red-shift in GR, though the red-shift is much smaller in GR (3 nm) than in PR (20 nm). Figure 2c shows that the second loop chimera (GR/Rh133-152) exhibits a 6-nm red shift, suggesting a similar mechanism of the third loop chimera. While the GR/Rh228-244 chimera shows *λ*_max_ at 542 nm, the double (GR/Rh133-152+228-244) loop chimera shows that *λ*_max_ was red-shifted by 7 nm (Figure 2d).

### G-Protein Activation Properties of the Third Loop Inserted into PR/Rh and GR/Rh Chimera

**Figure 3:**
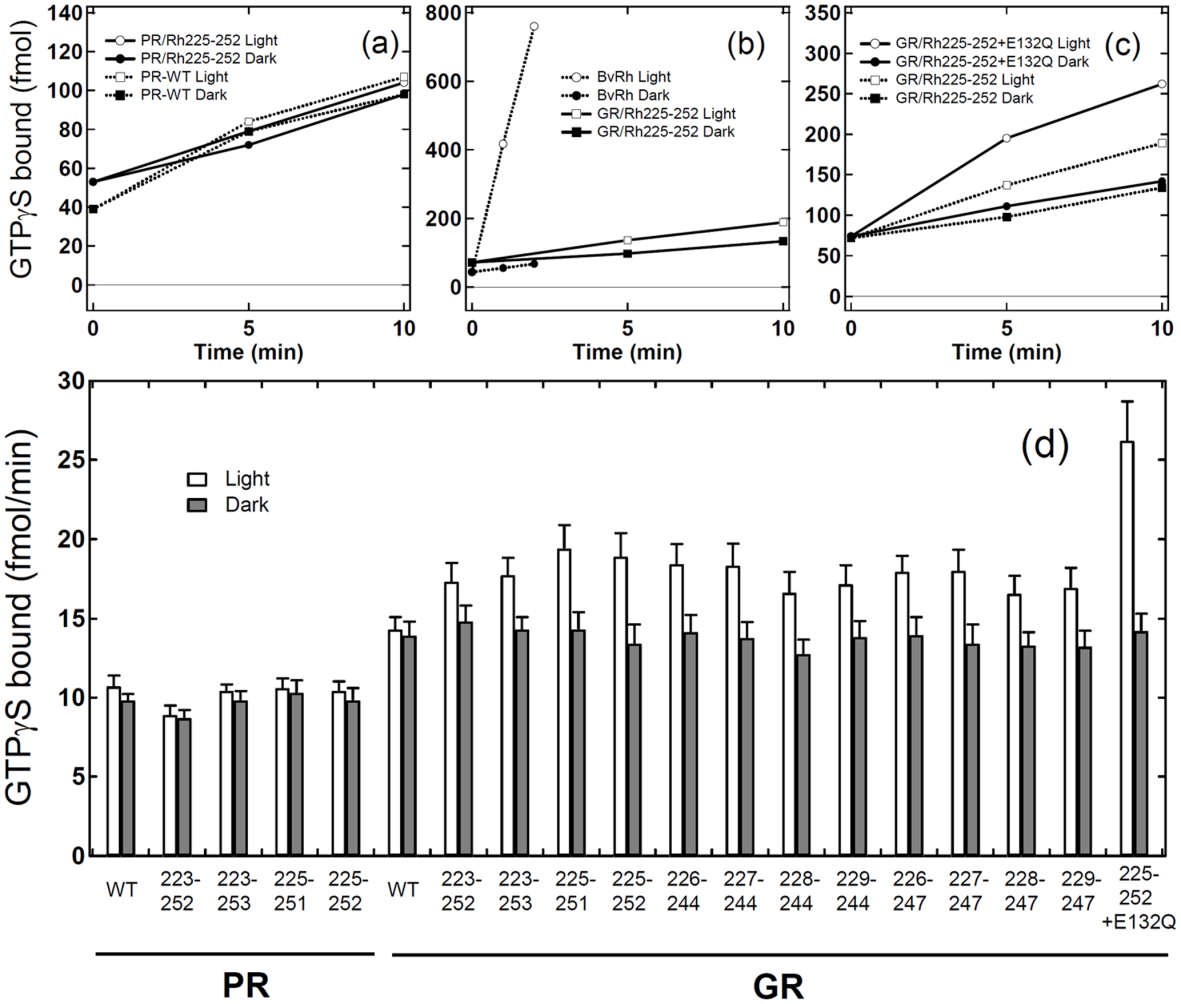
GTPγS-binding assay of the catalytic activities of third-loop inserted chimeras. G-protein activation by PR/Rh chimera (a), bovine Rh (b), and GR/Rh chimeras (c). Time-dependent GTPγS-binding to transducin was monitored under light (open circle or square) and dark (filled circle or square) conditions. Solid lines in (a) represent the results of PR/Rh225-252, while dotted lines in (a) represent WT PR. Solid lines in (b, c) represent the results of GR/Rh225-252 and GR/Rh225-252 + E132Q, while dotted lines in (b) and (c) represent bovine Rh and GR/Rh225-252, respectively. The concentrations of bovine Rh, PR (PR chimera) and GR (GR chimera) are 5 nM, 2.1 μM, and 1.9 μM, respectively. Binding at t = 0 was caused by unspecific binding to the filter and was not used to assay the catalytic activities of pigments. (d) Comparison of G-protein activation ability by PR/Rh and GR/Rh chimeras. GTPγS-binding to transducin was monitored at 10 min under light (open bar) and dark (filled bar) conditions. Data are presented as the means ± S.D. of more than three independent experiments.

We next tested the G-protein activation of PR/Rh and GR/Rh chimeras. Figure 3a-c shows the time-course of the binding of GTPγS to transducin, where the light-dependent GDP/GTPγS exchange was monitored with [^35^S]GTPγS. Because at least 200 DDM molecules are needed to solubilize one microbial rhodopsin [40], 0.05% DDM was used in the present study to fully solubilize the chimeric proteins whose concentration was *ca*. 2 μM (the molecular ratio of chimera : DDM is 1∶500). In PR/Rh225-252 (Figure 3a), the amount of light induced the time-dependent binding of GTPγS as much as the dark level of PR/Rh chimera, which was also the case for WT PR (dotted lines). There was also no light-dependent activation of PR/Rh223-252, PR/Rh223-253, and PR/Rh225-251. Thus, it can be concluded that PR/Rh chimeras do not activate G-protein.

Figure 3b shows the light-dependent G-protein activation of bovine Rh (dotted lines) and GR/Rh225-252 (solid lines). G-protein activation of GR/Rh225-252 was much weaker than for bovine Rh (Figure 3b) and the difference between dark and light is clear if the y axis is extended (dotted lines in Figure 3c). Therefore, GR/Rh chimeras can activate G-protein unlike PR/Rh chimeras. In total 12 GR/Rh chimeras that possess different lengths of the third loop were prepared. Figure 3d clearly shows that all chimeras possess the ability to activate G-protein. The catalytic activity of GR/Rh223-252 was relatively small, whereas other chimeras exhibited similar catalytic activities, ranging from 80 to 140 μmol/min/mol pigment (Table 1). Here, the small amount of GTP*γ*S-binding to Gt was observed in the dark. We previously reported similar amount of GTPγS-binding occurs even in the experiment with bovine rhodopsin which binds an 11-*cis* retinal as an inverse agonist and is shown to have no activity in the dark [41], [42]. Thus, this GTPγS-binding in the dark represents the intrinsic reaction of Gt itself which becomes relatively higher at room temperature, and the chimeras also does not activate Gt without light. The present results for GR chimeras resemble those for SRII chimeras [19]. Among the 12 chimeras, the greatest catalytic activity was obtained for GR/Rh225-252 (138 μmol/min/mol pigment). SRII/Rh225-252 also showed the greatest catalytic activity among 9 SRII chimeras tested [19].

**Table 1:** Light-dependent G-protein activation of the wild-type and GR chimeras.

Sample	Catalytic activity^a^ (μmol/min/mol pigment)
**Third loop inserted chimeras**	
GR wild-type	10±3
GR wild-type + E132Q	5±3
GR/Rh 223-252	63±5
GR/Rh 223-253	85±8
GR/Rh 225-251	128±10
GR/Rh 225-252	138±8
GR/Rh 226-244	107±5
GR/Rh 227-244	115±10
GR/Rh 228-244	97±10
GR/Rh 229-244	83±5
GR/Rh 226-247	100±3
GR/Rh 227-247	115 ±3
GR/Rh 228-247	82±7
GR/Rh 229-247	93±7
**Second loop inserted chimeras**	
GR/Rh 132-152	10±5
GR/Rh 133-152	9±5
GR/Rh 134-152	5±5
**Double loop inserted chimeras**	
GR/Rh 133-152 + 228-244	480±40
GR/Rh 133-152 + 228-244 + E132Q	990±120
GR/Rh 133-152 + 225-252 + E132Q	1070±130

a Light-dependent catalytic activity in the GR was calculated by differences in activities in the light and dark conditions as shown in Figure 3 and Figure 6.

**Table 2:** Time constants (*τ*_1/e_; ms) of the globally fitted time constants calculated from O decay (612 nm) and recovery (525 nm), at 25°C.

Sample	O_1_-decay (ms)	O_2_-decay (ms)	O-decay (ms)^a^
GR wild-type	16±1 (88%)	85±20 (12%)	24
Y223R252	14±2 (64%)	67±10 (36%)	33
Y223M253	15±1 (90%)	90±23 (10%)	22
Q225T251	22±2 (68%)	125±21 (32%)	55
Q225R252	23±2 (72%)	130±20 (28%)	53
L226Q244	11±1 (79%)	83±18 (21%)	27
V227Q244	29±3 (73%)	153±31 (27%)	63
F228Q244	17±1 (83%)	149±20 (17%)	40
T229Q244	21 ±1 (69%)	121±13 (31%)	52
L226E247	15±1 (80%)	130±15 (20%)	38
V227E247	36±2 (75%)	196±36 (25%)	76
F228E247	21 ±1 (82%)	182±34 (18%)	50
T229E247	25±1 (79%)	201±37 (21%)	62

a O decay was calculated by the following formula.

τO  =  ΔA1*τO1/(ΔA1 + ΔA2)+ ΔA2*τO2/(ΔA1 + ΔA2)

where ΔA i : Amplitude of the i-th O-decay, τOi: Time constant of the i-th O-decay.

Solid lines in Figure 3c show the results of GR/Rh225-252 + E132Q, whose catalytic activity (300 μmol/min/mol pigment) was more than 2-fold greater than that of GR/Rh225-252 (Figure 3d and Table 1). E132 in GR serves as the proton donor of the Schiff base, which corresponds to D96 in BR. Since Q132 does not act as the proton donor of the Schiff base, we expected a slower photocycle for E132Q, which may lead to larger G-protein activation. It seems that a long photocycle yields higher G-protein activation as is the case for the signaling state of bovine Rh (Meta-II) [6].

It is now possible to quantitatively compare the activation level between bovine Rh and GR/Rh chimeras. For the former, GTPγS binding was calculated to be 3.75 mol/min/mol pigment, using the molar extinction coefficient of bovine Rh (40,600 M^−1^cm^−1^) [43]. For the latter, we calculated the GTPγS binding of third loop inserted GR/Rh chimeras and the activation level ranged from 63×10^−6^ mol/min/mol pigment (GR/Rh223-252) to 138×10^−6^ mol/min/mol pigment (GR/Rh225-252), assuming that the molar extinction coefficients are identical to that of WT GR (50,000 M^−1^ cm^−1^) [44]. The most efficient chimera was GR/Rh225-252 (138×10^−6^ mol/min/mol pigment) in this study, and SRII/Rh225-252 (99×10^−6^ mol/min/mol pigment) in a previous report [19], indicating a similar tendency and slightly higher activation for the GR chimera. The activation level of the GR/Rh225-252 chimera was about 27,000 times lower than that of bovine Rh, while the E132Q mutant further increased the activation (12,500 times lower than bovine Rh).

### Photochemical Properties of the GR/Rh Chimera

Increased activation of GR/Rh225-252 + E132Q suggests a strong correlation between the photocycle dynamics and G-protein activation. However, a previous report did not provide clear correlation for the SRII chimera [19]. We thus studied the photocycle dynamics of GR/Rh chimeras. GR has unique cyclic reactions that are comprised of a series of intermediates, such as blue-shifted M and red-shifted O intermediates [25]. (All results of flash photolysis measurement are summarized in Figure S5 and S6)

**Figure 4:**
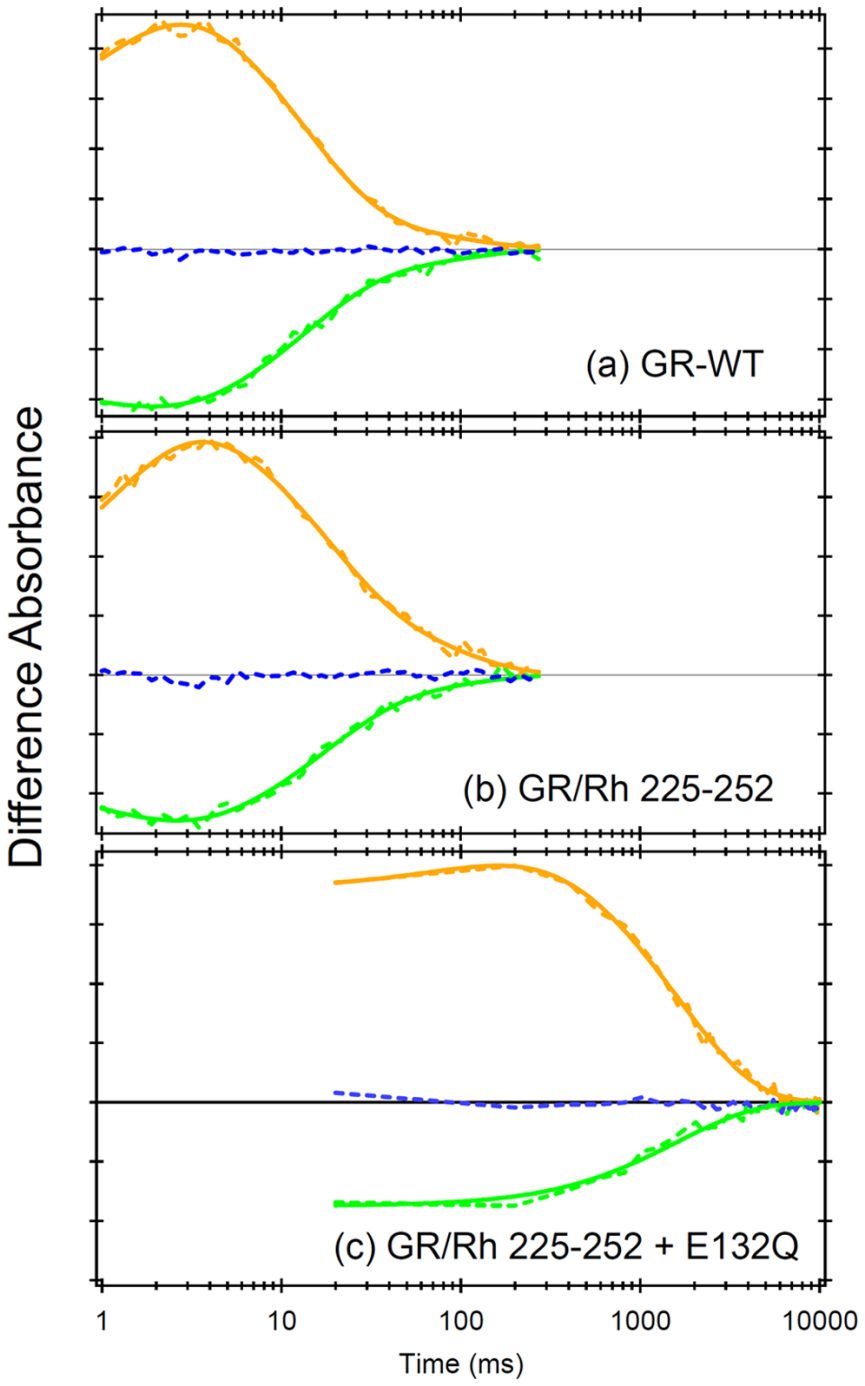
Laser flash photolysis results of GR at 25°C. Light-induced changes in absorbance of WT GR (a) and GR/Rh225-252 (b) were monitored at 400 nm (blue), 520 nm (green), and 615 nm (orange), which monitor the M intermediate, the depletion of GR, and the O intermediate, respectively. Solid broken lines represent the data points, which were averaged for 50 signals. Solid curved lines correspond to fitting curves (green; triple exponential, orange; triple exponential of rise and two components of O decay). (c) Light-induced absorbance changes of GR/Rh225-252 + E132Q were monitored at 400 nm (blue), 510 nm (green), and 610 nm (orange), which monitor the M intermediate, the depletion of GR, and the O intermediate, respectively. Solid broken lines represent the data points, which were averaged for 25 signals. Solid curved lines correspond to fitting curves (green; double exponential, orange; double exponential of rise and decay). All divisions on the y-axis correspond to 0.01 absorbance units.

Figure 4a shows laser-induced absorbance changes of WT GR at 400 nm (blue line), 525 nm (green line), and 612 nm (orange line), which monitor the M intermediate, recovery of GR, and the O intermediate, respectively. From this data, it seems that accumulation of the M intermediate was negligible and that the O intermediate is dominant in this time region. Therefore, we obtained the time constants (*τ*_1/e_) using multi-exponential curve fitting for the recovery and the O intermediate since two components were needed to describe the O decay, O_1_ and O_2_. The time constants of the O_1_ and O_2_ decay of WT GR were 16±1 ms and 85±20 ms (mean ± S.D.), respectively. The time constant of the O_1_ and O_2_ decay of GR/Rh225-252 (Figure 4b) was 23±2 ms and 130±20 ms, respectively. This suggests a slower O decay for the GR/Rh chimera. Figure 4c shows the data of GR/Rh225-252 + E132Q, the most efficient chimera. In this case, only one component was sufficient for the O decay, whose time constant was 1620±40 ms. Thus, the E132Q mutation yielded a long photocycle, which may be the reason for the increased activation of G-protein.

**Figure 5:**
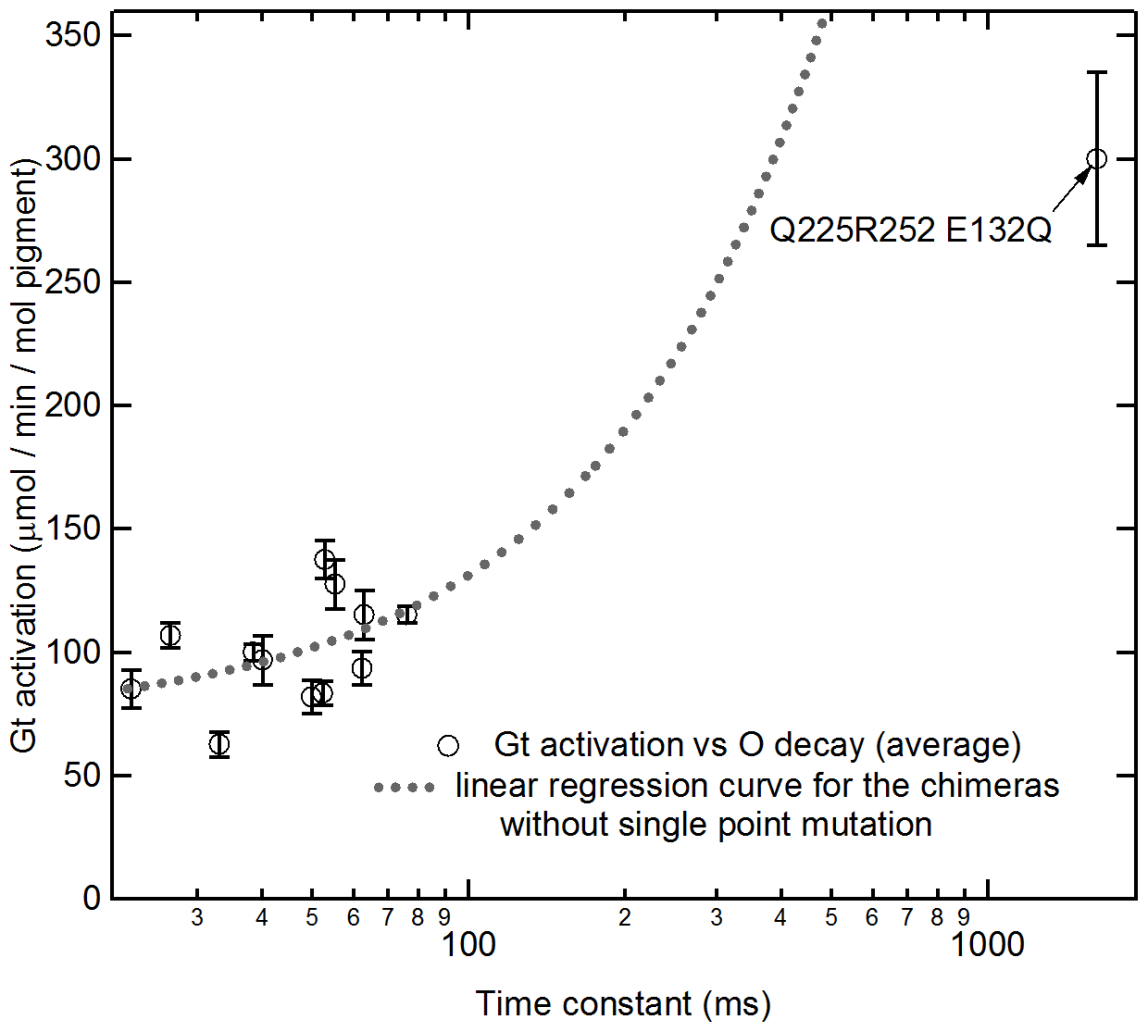
Correlation between the time constant of the decay of O intermediate of the bovine Rh third loop inserted GR chimera and the ability to activate G-protein. The time constant of O intermediate decay is calculated in Table 2. The value of G-protein activation ability was calculated using Figure 3c by subtracting GTPγS bound in the dark condition from GTPγS bound in the light condition as shown in Table 1. The dashed line is the linear regression curve for the data of chimeras without the E132Q mutation. Note that the horizontal axis is shown with a logarithmic scale. Data are presented as the means ± S.D. of more than three independent experiments.

We next examined the relationship between time constants and G-protein activation among the 12 GR/Rh chimeras. These GR/Rh chimeras showed unique G-protein activation (Figure 3) and O decay (Table 1). Figure S7 shows the correlation between G-protein activation and time constant of the O intermediate, in which we evaluated Pearson's correlation coefficient (*r* value). The *r* value ranged from minus one to plus one (−1≤*r*≤1) and “minus” and “plus” indicate negative and positive correlations, respectively. There was no significant correlation between G-protein activation and the time constant of the O intermediate (*r* = 0.44; Figure S7). The higher catalytic activity is expected for the longer decay rate, because the frequency of the interaction between the active state and G-protein increases. However, this result indicates small prolongation of decay rate does not enhance the activation efficiency so much and other factors cause the variation of the catalytic activity. If a linear regression curve for the data of the chimeras without the E132Q mutation is calculated and extended to a longer time-constant region, the catalytic activity of GR/Rh225-252 + E132Q is much lower than the value expected form the curve (Figure 5). This discrepancy would arise from the saturated accumulation of photo-product that occurs when the lifetime is longer. Actually, we estimated the amount of the accumulation of O intermediate in our G-protein activation assay experiment by numerical calculation (the detailed description of the calculation is shown in Document S1) and the result is shown in Figure S8. It shows that if the lifetime of O is faster than 100 ms, the amount of O accumulation linearly increases with the enhancement of the lifetime. On the other hand, if the lifetime is >1000 ms, most of molecule (>95%) is converted to O and further enhancement by the prolongation of lifetime does not occur. This explains the lower enhancement of catalytic activity by E132Q mutation compared with the value expected by the prolongation of the decay rate. Thus, we suggest further elongation of the lifetime of O-intermediates would thus not enhance G-protein activation.

### Proton-Pump Activity of GR/Rh Chimera

**Figure 6:**
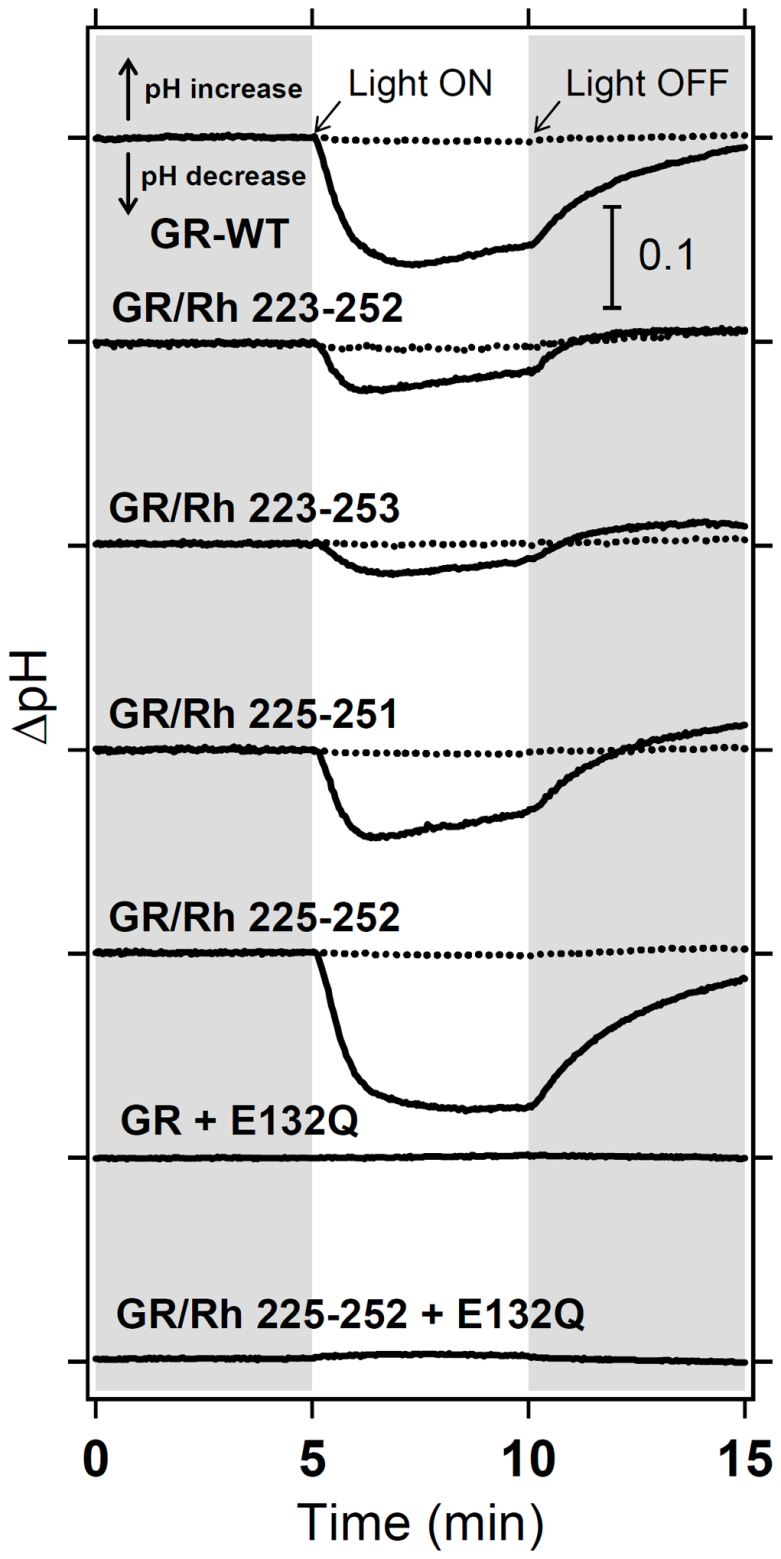
Proton pump activities of the GR/Rh chimeras. Proton pump activity of WT GR, GR/Rh 223-252, GR/Rh 223-253, GR/Rh 225-251, GR/Rh 225-252, GR E132Q, and GR/Rh 225-252 + E132Q (solid lines). After the addition of CCCP, all light-induced decreases in pH disappeared (dotted lines).

The GR/Rh chimeras activate G-protein, indicating that a new function was gained. But do the GR/Rh chimeras pump protons? Figure 6 compares light-driven proton pump activity of WT and chimeric GR. In the case of WT GR, illumination caused a net acidification of the medium (solid curve), indicating that protons are pumped out of the *E. coli* cell. The addition of 10 μM CCCP almost abolishes the observed light-induced change in pH (dotted line). Similar results were obtained for GR chimeras, although pumping activities differed among samples. In contrast, the E132Q mutant showed no pumping signals. The corresponding BR (D96N) mutant also exhibited no apparent proton pump signals detectable by a pH electrode. However, a proton release-reuptake process was observed as a change in absorption by the pH-indicator dye after illumination, allowing BR D96N to pump protons [45]. No apparent proton pump signal has been explained by a slow photocycle. Since the E132Q mutant of GR also shows a slow photocycle (Figure 4), the ability of E132Q mutants to pump protons is not excluded.

### G-Protein Activation Properties of the Double Loop Inserted GR/Rh Chimeras

Previously, we introduced the third cytoplasmic loop of bovine Rh into BR and SRII, and observed light-dependent Gt activation [19]. Nevertheless, the highest activation was only 1/30,000 (SRII/Rh225-252) compared to bovine Rh. The present study successfully reproduced the activation of Gt by the GR chimera, although the level of activation was still low. A higher level of activation is a basic necessity for optogenetic applications, and this possibility was further examined by additional mutations.

**Figure 7:**
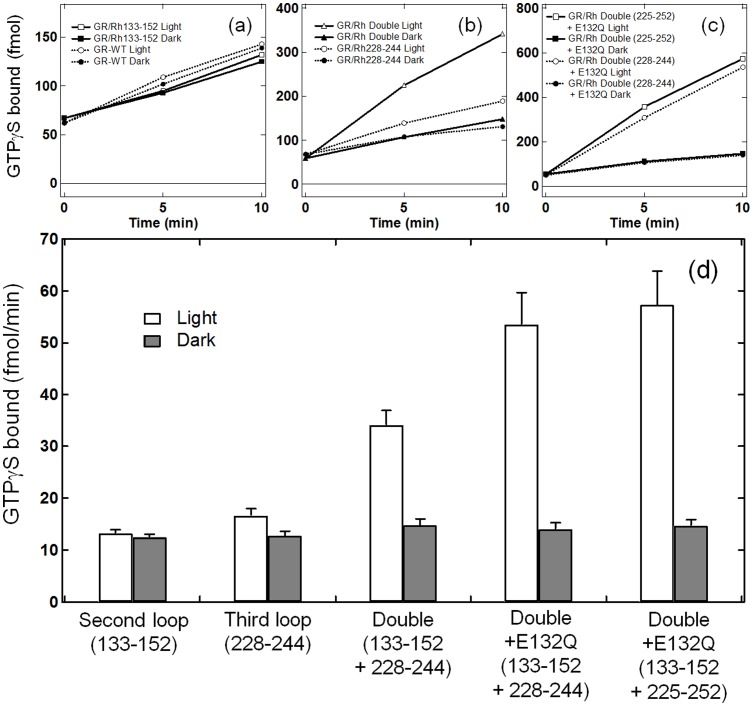
GTPγS-binding assay of the catalytic activities of second-loop and double-loop inserted chimeras. G-protein activation by the bovine Rh second loop inserted GR chimera (a), the double loop inserted GR chimera (b), and the E132Q-induced double loop inserted chimeras (c). Time-dependent GTPγS-binding to transducin was monitored under light (open circle or square) and dark (filled circle or square) conditions. Solid lines in (a) represent the results of GR/Rh133-152, while dotted lines in (a) represent WT GR. Solid lines in (b) represent the results of GR/Rh133-152 + 228-244, while dotted lines in (b) represent the result of GR/Rh228-244. Solid lines in (c) represent the results of GR/Rh133-152 + 225-252 + E132Q, while dotted lines in (c) represent the results of GR/Rh133-152 + 228-244 + E132Q. (d) Comparison of G-protein activation ability by GR/Rh chimeras. GTPγS-binding to transducin was monitored at 10 min under light (open bar) and dark (filled bar) conditions. Data are presented as the means ± S.D. of more than three independent experiments.

It is known that the second cytoplasmic loop plays an important role in the receptor-transducer interaction, which has a highly conserved ERY motif in the type A GPCR family [6], [32]. Therefore, we prepared GR/Rh133-152 that possesses the second cytoplasmic loop of bovine Rh. While BR chimera containing the second cytoplasmic loop of bovine Rh did not turn purple and might not bind retinal [18], GR/Rh133-152 was successfully expressed and purified with a 7.6-fold lower yield. Figure 7a shows that GR/Rh133-152 and WT GR exhibit no light-dependent activation of G-protein. Even though the second loop chimera was not activated, further experiments provided interesting observations. Figure 6b compares the light-dependent activation between the third loop chimera (GR/Rh228-244) and second/third loop chimera (GR/Rh133-152 + 228-244) in which the activation of G-protein is about five times higher for the double loop chimera. This result clearly demonstrates that introduction of the second loop is not sufficient to activate G-protein, but that the second loop obviously contributes to increase the efficiency. The efficiency of this chimera is 7,800 times lower than that of bovine Rhs. Figure 6c and d show that activation by the double loop of G-protein is further promoted about two-fold by a mutation, E132Q. A two-fold higher activation by E132Q also took place for single third loop chimeras (Figure 3). From Figure 6d, the activation of G-protein by GR/Rh133-152 + 228-244 + E132Q and by GR/Rh133-152 + 225-252 + E132Q was 3,500 and 3,200 times lower, respectively than the activation of bovine Rh. The values are still much lower than the native system. It should be noted however that bovine Rh has the most efficient light-dependent activation of G-protein because twilight vision needs high efficiency. For example, Go-rhodopsin, an ancestral visual pigment, is activated 50 times less than bovine Rh [7]. Therefore, the activation efficiency of the present chimera, GR/Rh133-152 + 225-252 + E132Q, is only 64 times lower than that of Go-rhodopsin.

## Discussion

### Properties of Chimeras

In the present study, we prepared the chimeric proteins of PR and GR containing the third loop of bovine Rh. The PR chimeras showed a 20-nm red shifted absorption as well as an increase in the pKa of the Schiff base counterion. This may sounds unusual because the replaced loop is distant from the chromophore region. Nevertheless, we previously reported that the A178X mutant in the third loop of PR caused a 20-nm red shift in absorption and increased the pKa of the Schiff base counterion [38], [39], which was fully reproduced in this study. We found an unusual volume-dependent change in color of position 178, suggesting that the equilibrium of both PR protein conformations is dependent on the amino acid at that position. Since the replaced loop of PR includes this region, the bovine Rh loop presumably alters the equilibrium of the protein conformations. Similar observations were made for the GR chimera, although the spectral shift was much smaller. In the case of GR chimeras, the spectral red-shift was larger for the second loop (6 nm) than for the third loop (3 nm). These results suggest that the distant mutation effect is common among eubacterial rhodopsins and it is useful in optogenetic application to construct new rhodopsins working with longer-wavelength light which is less toxic for the cells. In addition, this long-range effect on the absorption wavelength of retinal provide new basis for the comprehensive understanding of the color-tuning mechanism by biophysical and chemical studies [46]–[48]. Some theoretical studies estimated the effect of the residues distant from retinal on its absorption maximum and explained the mechanism [49]. However, ∼20-nm red-shifts observed for PR chimeras are much higher than the previous theoretical studies for long-range color tuning mechanism, and there are no theory reported which can explain why the amount of the shift depends on the volume of the side chain on this position. Thus, our findings need to be studied furthermore for comprehensive understanding of color tuning mechanism.

### G-Protein Activation Mechanism by Chimeras

The present study shows that all GR chimeras containing the third cytoplasmic loop activated G-protein, as previously reported for BR and SRII [19], while PR chimeras did not (Figure 3). These results may originate from different conformational changes at the cytoplasmic surface. In the case of bovine Rh, outward movement of helix 6 (F-helix) occurs in Meta-II, which is a prerequisite for G-protein activation. Movement of helix 6 by 6–7 Å between the dark and peptide-bound opsin states, the latter mimicking the active state, was reported by X-ray crystallography [50], which was also supported by spin-labeling [51] and an engineered metal-ion-binding study [52]. A similar helix opening motion (F-helix) was reported for BR from cryo-electron microscopy [53], X-ray diffraction [54], [55], neutron diffraction [56], spin labeling [57], and high-speed atomic force microscopy [58]. A spin-labeling study also showed that the F-helix opens SRII [59]. Therefore, G-protein activation by BR and SRII chimeras in a previous study [19] can be interpreted as a similar helix opening motion between visual and microbial rhodopsins.

**Figure 8:**
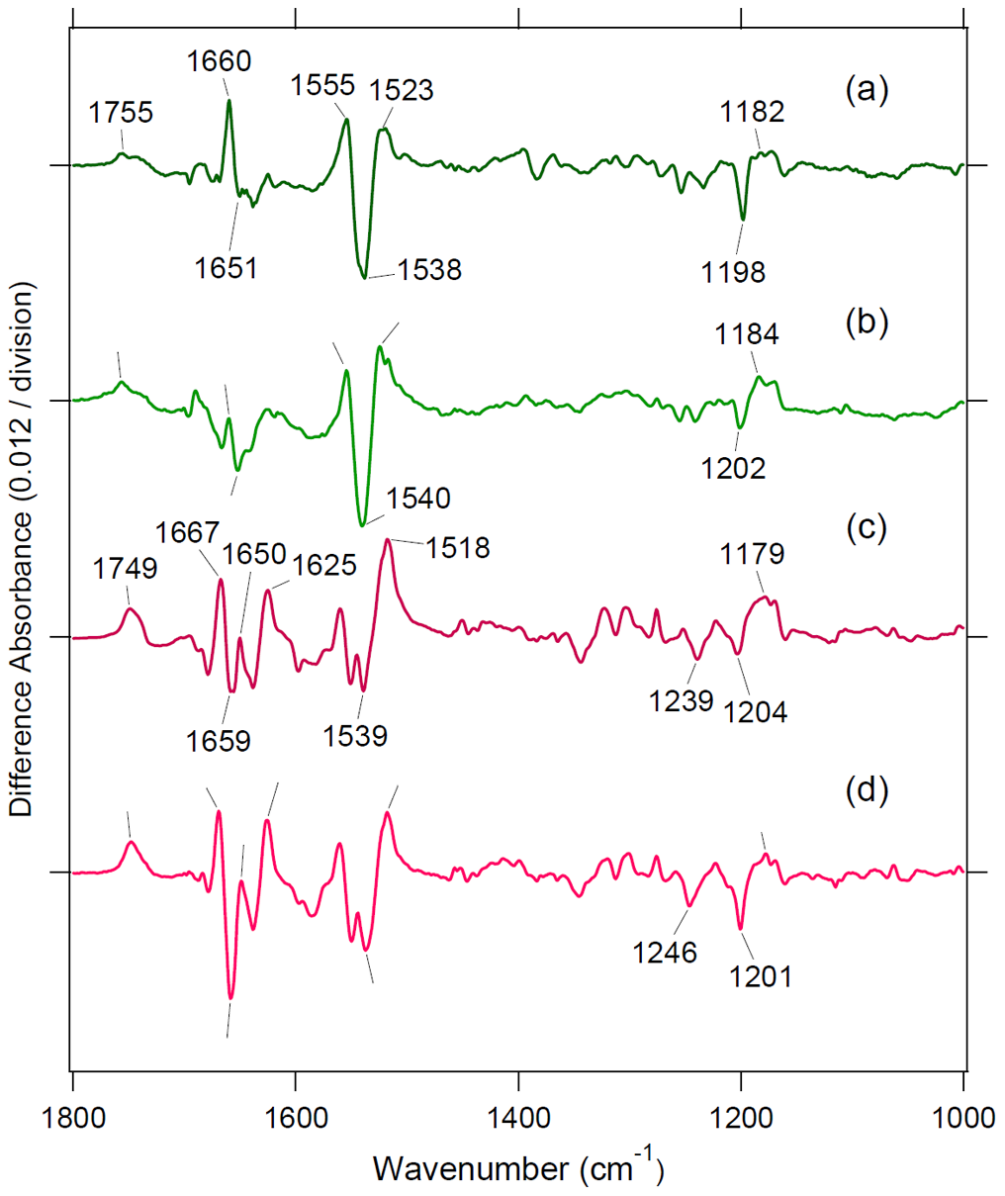
Low-temperature light-induced FTIR spectroscopy. Light-induced difference FTIR spectra of WT PR (a), PR/Rh225-252 (b), WT GR (c), and GR/Rh113-152 + 225-252 + E132Q in the 1800-950 cm^−1^ region. Each sample was illuminated with a 520±5 nm light at 250 K and pH 7.0. Positive and negative bands originate from photointermediate and unphotolyzed states, respectively.

G-protein activation by GR chimeras can be similarly interpreted by helix opening on the cytoplasmic side, though there are no reports for GR. If this is the case, the lack of G-protein activation by PR chimeras suggests no helix opening on the cytoplasmic side for PR. Therefore, we tested this hypothesis by using light-induced difference FTIR spectroscopy. Figure 8 compares structural changes of PR, GR, and their chimeras, in which the amplitude of chromophore structural changes was roughly normalized by retinal vibrations (C-C stretch at 1250-1100 cm^−1^). By doing so, helical structural perturbations can be monitored using the amide-I vibration at 1660-1650 cm^-1^. In PR, a peak pair at 1660 (+)/1651 (−) cm^−1^ is characteristic of the structural perturbation of α-helices, where the signal of the chimera (Figure 8b) was smaller than that of WT (Figure 8a). On the other hand, the FTIR spectra of WT GR (Figure 8c) and the chimera (Figure 8d) showed lower shifts of retinal C = C stretching bands (1539(−)/1518(+) cm^−1^). These lower shifts are characteristic of the O-intermediate [25] and indicate that O dominantly accumulates in identical continuous light illumination in the GTPγS-binding assay. The bands at 1667 (+)/1659 (−)/1650 (+) cm^−1^ can be interpreted as a structural perturbation of α-helices in GR in which the chimera (Figure 8d) shows a larger signal than WT (Figure 8c). We infer that the helix opening motion on the cytoplasmic side takes place similarly in BR, GR and PR because such a motion is a prerequisite for the formation of the proton pathway in the hydrophobic cytoplasmic domain. The present FTIR study suggests that small protein structural changes in the PR chimeras are the main reason for the lack of G-protein activation.

Using a site-directed fluorescence labeling approach, Tsukamoto et al. showed that the relative movement of helices 5 and 6 upon photoactivation was much greater in bovine Rh than in parapinopsin, a non-visual rhodopsin [60], which explains the 20-fold more effective G-protein activation of bovine Rh than of parapinopsin [7]. Tsukamoto et al. concluded that the different amplitudes of the helix movement were responsible for the functional diversity of G-protein-coupled receptors. This conclusion may be applicable to the chimeric microbial rhodopsins. If GR and SRII chimeras of the same design are compared, GR/Rh225-252 has about a 30 times faster photocycle than SRII/Rh225-252, but the efficiency of G-protein activation is higher in GR (GR: 137 μmol/min/mol pigment, SRII: 99 μmol/min/mol pigment). Therefore, larger conformational changes may occur in the O intermediate of GR, relative to SRII in which the M intermediate is considered to be an active state. GR chimeras show no G-protein activation in the dark, suggesting that the interaction surface of the receptor is hidden in the dark and only exposed by light, as well as bovine Rh.

Another interesting observation is the correlation between G-protein activation and the lifetime of the O intermediate, though the correlation was weak (*r* = 0.44). This suggests that the O intermediate is responsible for G-protein activation. In other words, the O intermediate is responsible for the helix opening motion at the cytoplasmic surface. It is well established that M and N intermediates are responsible for helix opening in BR and SRII, which may be contradicted by the present results. In fact, the present photocycle measurements showed no accumulation of the M intermediate in GR chimeras (Figure 4) in our experimental condition at pH 7. However, this is not unusual because it was suggested that the O intermediate of GR possesses 13-*cis* retinal. A recent study on *Exiguobacterium sibiricum* rhodopsin also suggested that the O intermediate possesses 13-*cis* retinal, which can be a common property of eubacterial rhodopsins [61]. Figure 8c and d show that the C-C stretching frequency of the O intermediates are similar (1179 cm^−1^) for WT and the GR chimera, suggesting that the O intermediate of GR chimeras has 13-*cis* retinal. It is thus likely that the O intermediate has a red-shifted absorption for GR and GR chimeras (thus called “O”) while the chromophore structure is a 13-*cis* form (more “N-like”).

Among the third-loop inserted GR chimeras, the most effective protein was GR/Rh225-252, which was also the case for SRII chimeras. Then, we prepared eight kinds of chimeras possessing different lengths and orientations (from 226, 227, 228, 229 to 244, 248). The results showed some variation in which the chimera starting from position 227 had the highest catalytic activity. This fact also indicates the importance of position, in which the interaction with the G-protein differs. To better design a chimera, such factors should be taken into account. In addition, second-loop inserted GR chimeras showed no G-protein activation, whereas the double loop inserted GR chimera activated G-protein about five times more than the corresponding third-loop inserted GR chimera. This fact clearly shows the importance of the second loop, consistent with previous results [32].

The GR/Rh133-152 + 225-252 + E132Q chimera activates G-protein 3,200 times less than bovine Rh. This level seems to be too low to use as an optogenetic tool. However, if the activation with parapinopsin and Go interacting pigment of invertebrate animals is compared, the G-protein activation of the best chimera is 160- and 64-fold lower, respectively [7]. This level of high activation suggests that GR chimeras have potential to create new optogenetic tools. To achieve this, it is important to design proteins that can activate more general G-proteins such as Gs, Gi, Go, etc. Another topic of interest would be the study of intracellular catalytic activity of new chimeras coupled to other G-proteins. Insertion of such loops is now in progress. At that stage, the selectivity of G-proteins catalyzed by chimeras would be important. We are planning to use the cytoplasmic loops of GPCRs for specific coupling with each G-protein. The improved level of activation is an important objective that needs to be achieved.

## Supporting Information

Figure S1 **Alignment of amino-acid sequences of BR** [**18]**, [19] **, SRII** [**19**] **, PR and GR chimeras.** The positions of transmembrane helices are based on the crystal structure of BR (1BM1) [62].
(TIF)

Figure S2 **Absorption spectra of PR/Rh chimeras.** PR/Rh223-252 (solid line in (a)), PR/Rh223-253 (solid line in (b)), PR/Rh225-251 (solid line in (c)), PR/Rh225-252 (solid line in (d)). Broken lines are the absorption spectrum of wild-type PR. One division of the y-axis corresponds to 0.2 absorbance units. All samples were solubilized in 0.1% DDM solution.
(TIF)

Figure S3 **The absorption maxima of PR/Rh chimeras.** PR/Rh223-252 (a), PR/Rh223-253 (b), PR/Rh225-251 (c), and PR/Rh225-252 (d) at various pHs which were fitted by the Henderson-Hasselbalch equation shown by a solid line. The absorption maximum of wild-type-PR is shown and was fitted by the Henderson-Hasselbalch equation, shown by a broken line in each panel.
(TIF)

Figure S4 **Absorption spectra of GR/Rh chimeras.** GR/Rh223-252 (a), GR/Rh223-253 (b), GR/Rh225-251 (c), GR/Rh225-252 (d), GR/Rh226-244 (e), GR/Rh227-244 (f), GR/Rh228-244 (g), GR/Rh229-244 (h), GR/Rh226-247 (i), GR/Rh227-247 (j), GR/Rh228-247 (k), GR/Rh229-247 (l), GR/Rh225-252 + E132Q (m), GR/Rh132-152 (n), GR/Rh133-152 (o), GR/Rh134-152 (p), GR/Rh133-152 + 228-244 (q), GR/Rh133-152 + 228-244 + E132Q (r), GR/Rh133-152 + 225-252 + E132Q (s), and GR/Rh133-152 + E132Q (t) (solid lines). Broken lines are the absorption spectrum of wild-type GR. One division of the y-axis corresponds to 0.2 absorbance units. All samples were solubilized in 0.1% DDM solution.
(TIF)

Figure S5 **Transient absorption spectra of GR/Rh chimeras.** GR/Rh223-252 (a), GR/Rh223-253 (b), GR/Rh225-251 (c), GR/Rh225-252 (d), GR/Rh226-244 (e), GR/Rh227-244 (f), GR/Rh228-244 (g), GR/Rh229-244 (g), GR/Rh226-247 (i), GR/Rh227-247 (j), GR/Rh228-247 (k), and GR/Rh229-247 (l). One division of the y-axis corresponds to 0.01 absorbance units. All samples were solubilized in 0.1% DDM solution.
(TIF)

Figure S6 **Laser flash photolysis results of GR/Rh chimeras at 25°C.** Light-induced absorbance changes of GR/Rh223-252 (a), GR/Rh223-253 (b), GR/Rh225-251 (c), GR/Rh225-252 (d), GR/Rh226-244 (e), GR/Rh227-244 (f), GR/Rh228-244 (g), GR/Rh229-244 (g), GR/Rh226-247 (i), GR/Rh227-247 (j), GR/Rh228-247 (k), and GR/Rh229-247 (l) monitored at 400 nm (blue), 520 nm (green), and 615 nm (orange), which indicate the M intermediate accumulation, the depletion of GR, and the accumulation of O intermediate, respectively. Solid lines with small noise represent the data points, which were averaged for 50 signals. Smooth solid lines correspond to fitting curves (green; triple exponential, orange; triple exponential of rise and two components of O decay). All divisions in the y-axis correspond to 0.01 absorbance units.
(TIF)

Figure S7 **Correlation between the time constant of the decay of O intermediate of bovine Rh third loop inserted GR chimera and the G-protein activation ability.** The time constant of the O intermediate decay was calculated in Table 2. The value of G-protein activation ability was calculated using Figure 3c by subtracting [^35^S]GTPγS bound in dark condition from GTPγS bound in light condition as shown in Table 1. Data are presented as the means ± S.D. of more than three independent experiments.
(TIF)

Figure S8 **The estimated correlation between the amounts of the accumulated O intermediate in G-protein activation assay with the decay rate of the intermediate (solid line).** Red circles represent the values for the mutants constructed in this study. The way of calculation is described in Document S1.
(TIF)

Document S1 **The way of the calculation of the estimation of the amounts of the accumulated O intermediate in G-protein activation assay is explained.**
(DOC)
