# Supplementary figures and images for: Chimeric Proton-Pumping Rhodopsins Containing the Cytoplasmic Loop of Bovine Rhodopsin
Source: PLoS One. 2014 Mar 12;9(3):e91323. doi: 10.1371/journal.pone.0091323 (PMC3951393; doi:10.1371/journal.pone.0091323)

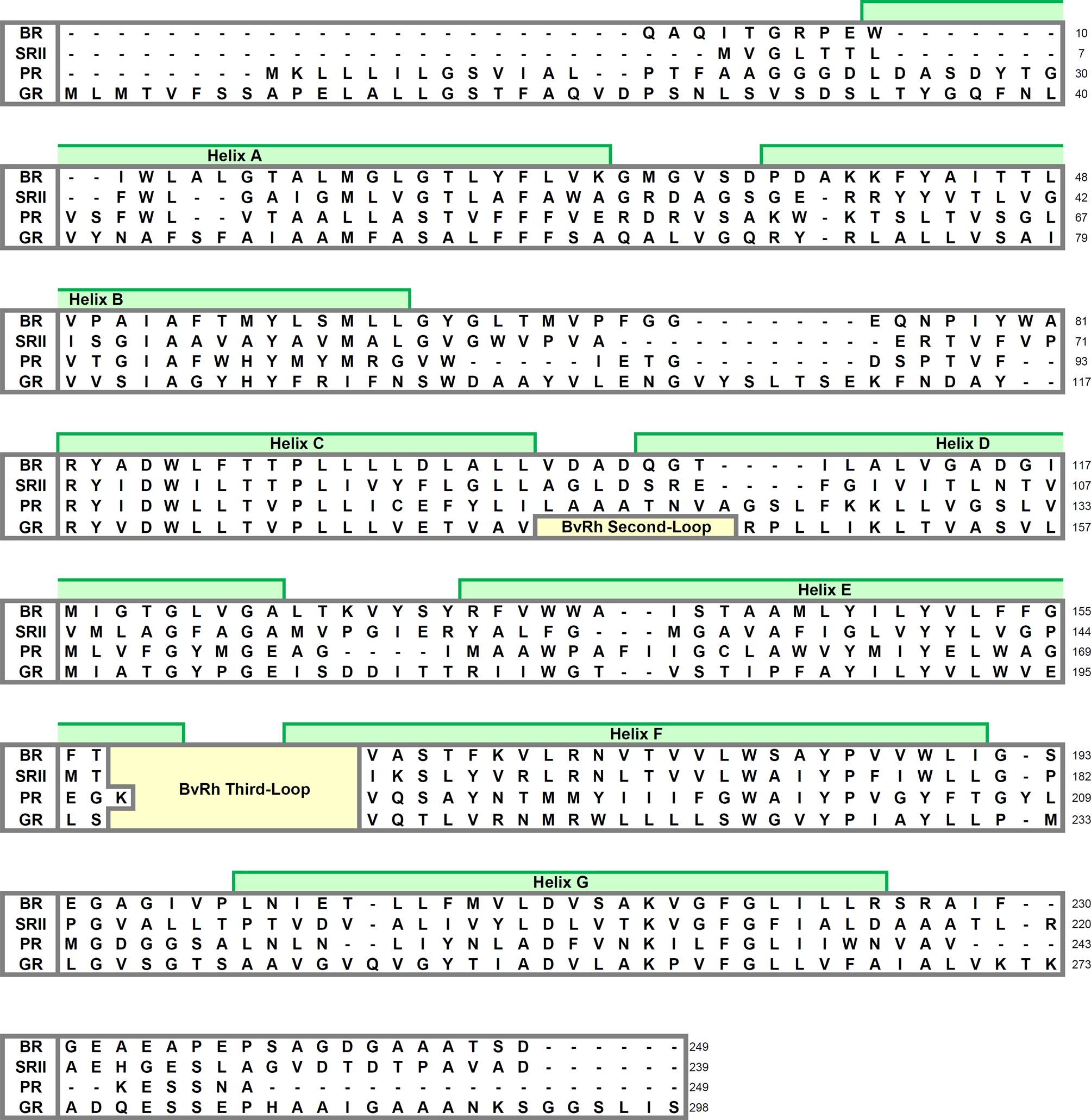

Supplement: Figure S1 — Alignment of amino-acid sequences of BR [18] , [19] , SRII [19] , PR and GR chimeras. The positions of transmembrane helices are based on the crystal structure of BR (1BM1) [62]. (TIF) [file pone.0091323.s001.tif]

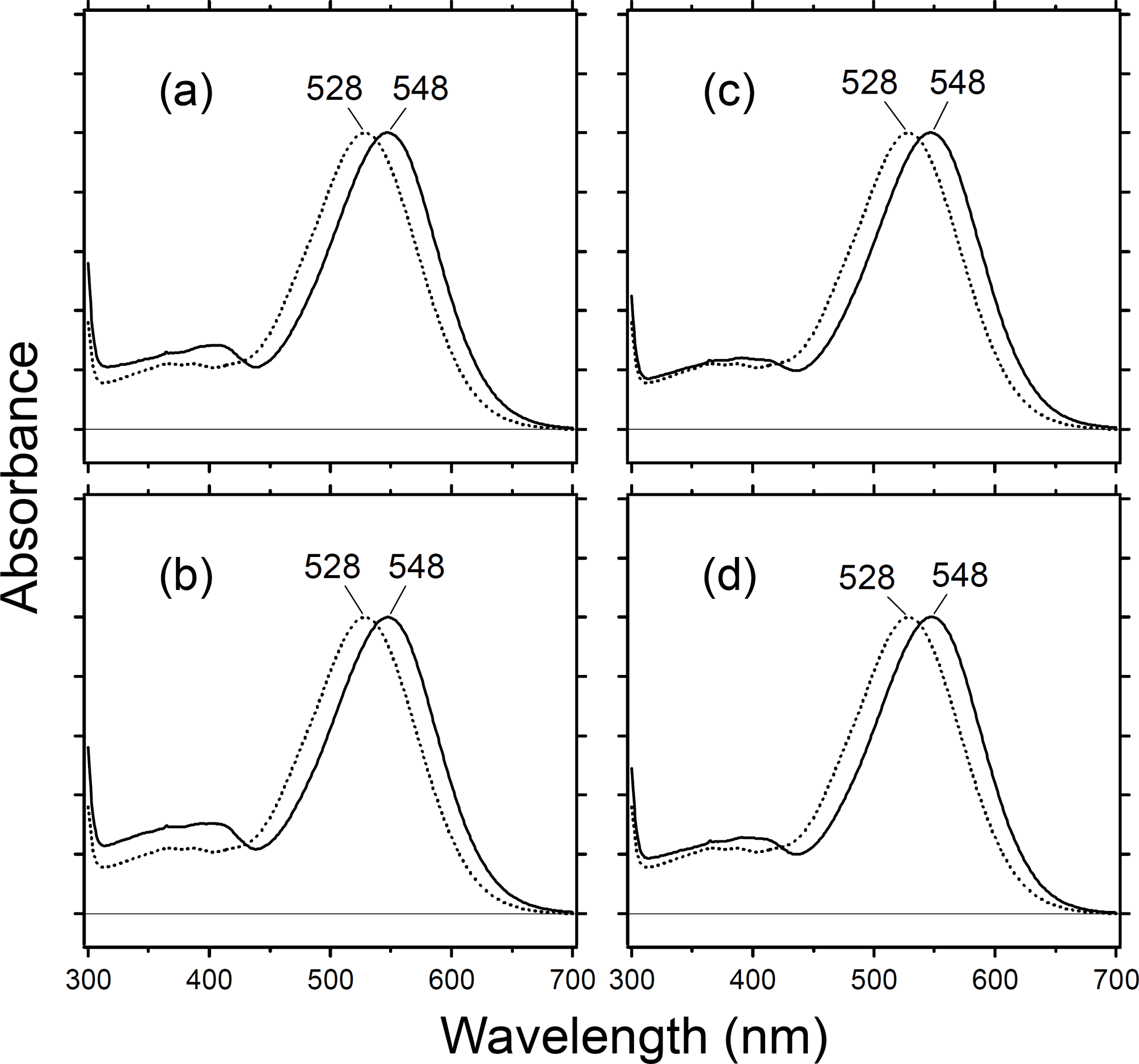

Supplement: Figure S2 — Absorption spectra of PR/Rh chimeras. PR/Rh223-252 (solid line in (a)), PR/Rh223-253 (solid line in (b)), PR/Rh225-251 (solid line in (c)), PR/Rh225-252 (solid line in (d)). Broken lines are the absorption spectrum of wild-type PR. One division of the y-axis corresponds to 0.2 absorbance units. All samples were solubilized in 0.1% DDM solution. (TIF) [file pone.0091323.s002.tif]

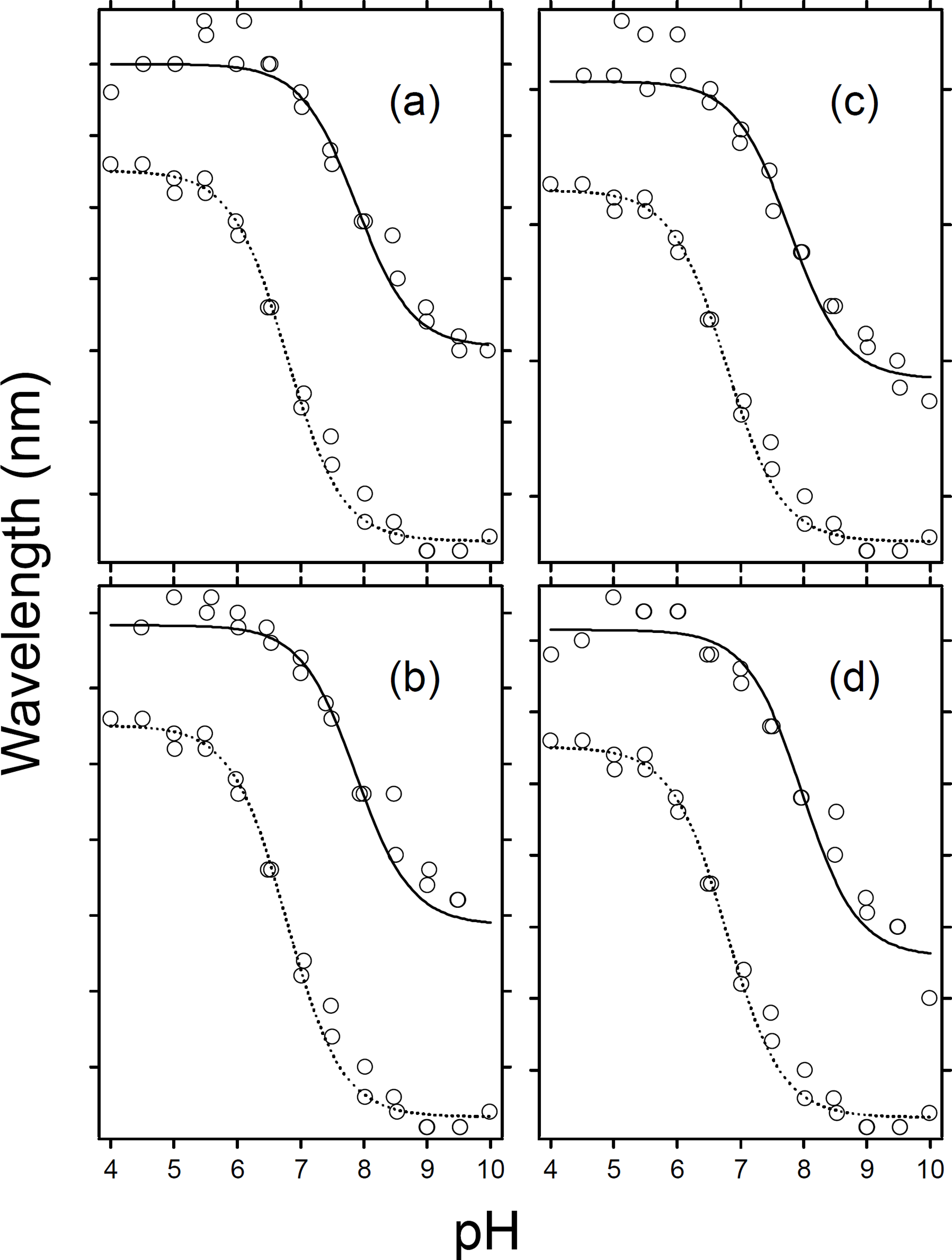

Supplement: Figure S3 — The absorption maxima of PR/Rh chimeras. PR/Rh223-252 (a), PR/Rh223-253 (b), PR/Rh225-251 (c), and PR/Rh225-252 (d) at various pHs which were fitted by the Henderson-Hasselbalch equation shown by a solid line. The absorption maximum of wild-type-PR is shown and was fitted by the Henderson-Hasselbalch equation, shown by a broken line in each panel. (TIF) [file pone.0091323.s003.tif]

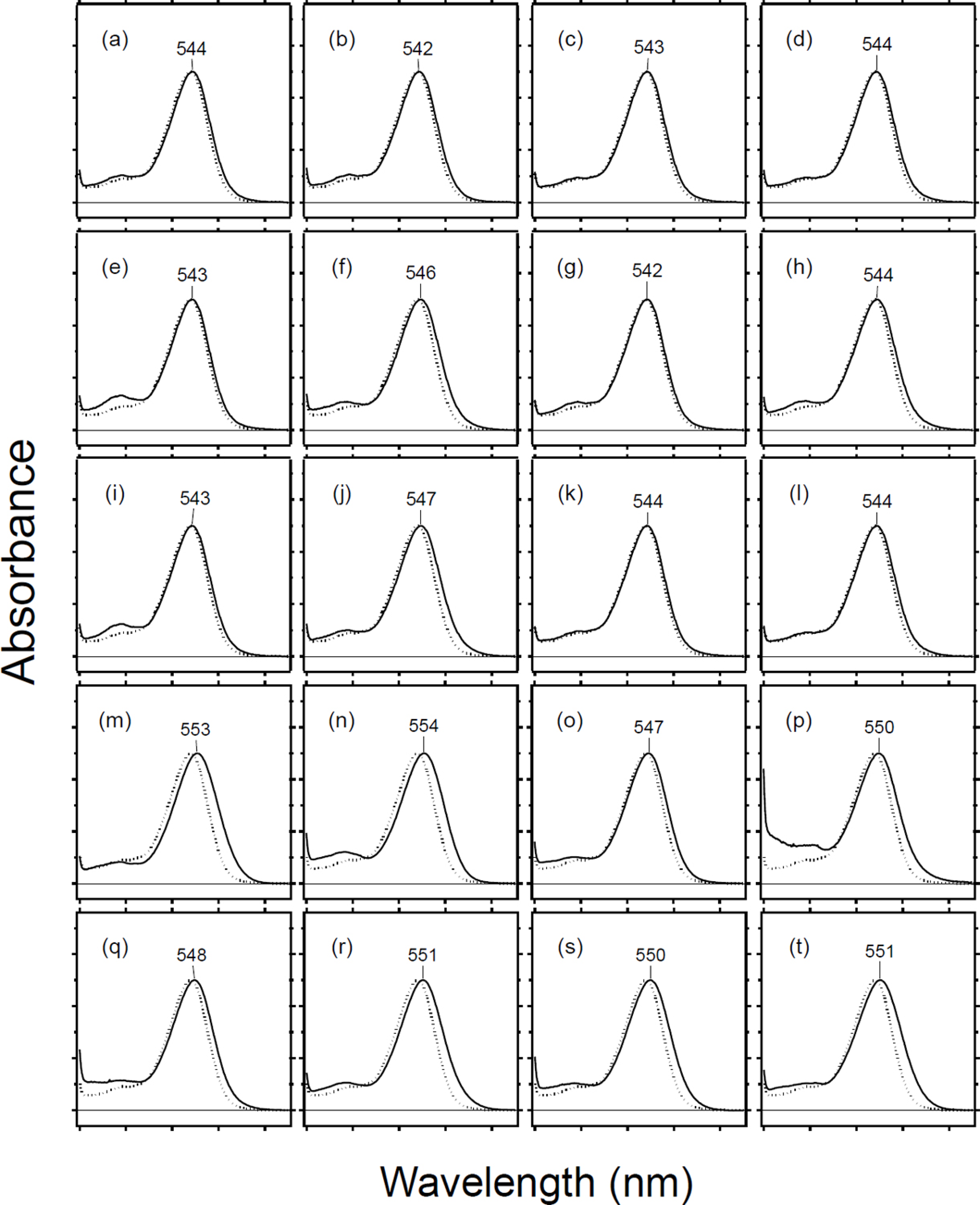

Supplement: Figure S4 — Absorption spectra of GR/Rh chimeras. GR/Rh223-252 (a), GR/Rh223-253 (b), GR/Rh225-251 (c), GR/Rh225-252 (d), GR/Rh226-244 (e), GR/Rh227-244 (f), GR/Rh228-244 (g), GR/Rh229-244 (h), GR/Rh226-247 (i), GR/Rh227-247 (j), GR/Rh228-247 (k), GR/Rh229-247 (l), GR/Rh225-252 + E132Q (m), GR/Rh132-152 (n), GR/Rh133-152 (o), GR/Rh134-152 (p), GR/Rh133-152 + 228-244 (q), GR/Rh133-152 + 228-244 + E132Q (r), GR/Rh133-152 + 225-252 + E132Q (s), and GR/Rh133-152 + E132Q (t) (solid lines). Broken lines are the absorption spectrum of wild-type GR. One division of the y-axis corresponds to 0.2 absorbance units. All samples were solubilized in 0.1% DDM solution. (TIF) [file pone.0091323.s004.tif]

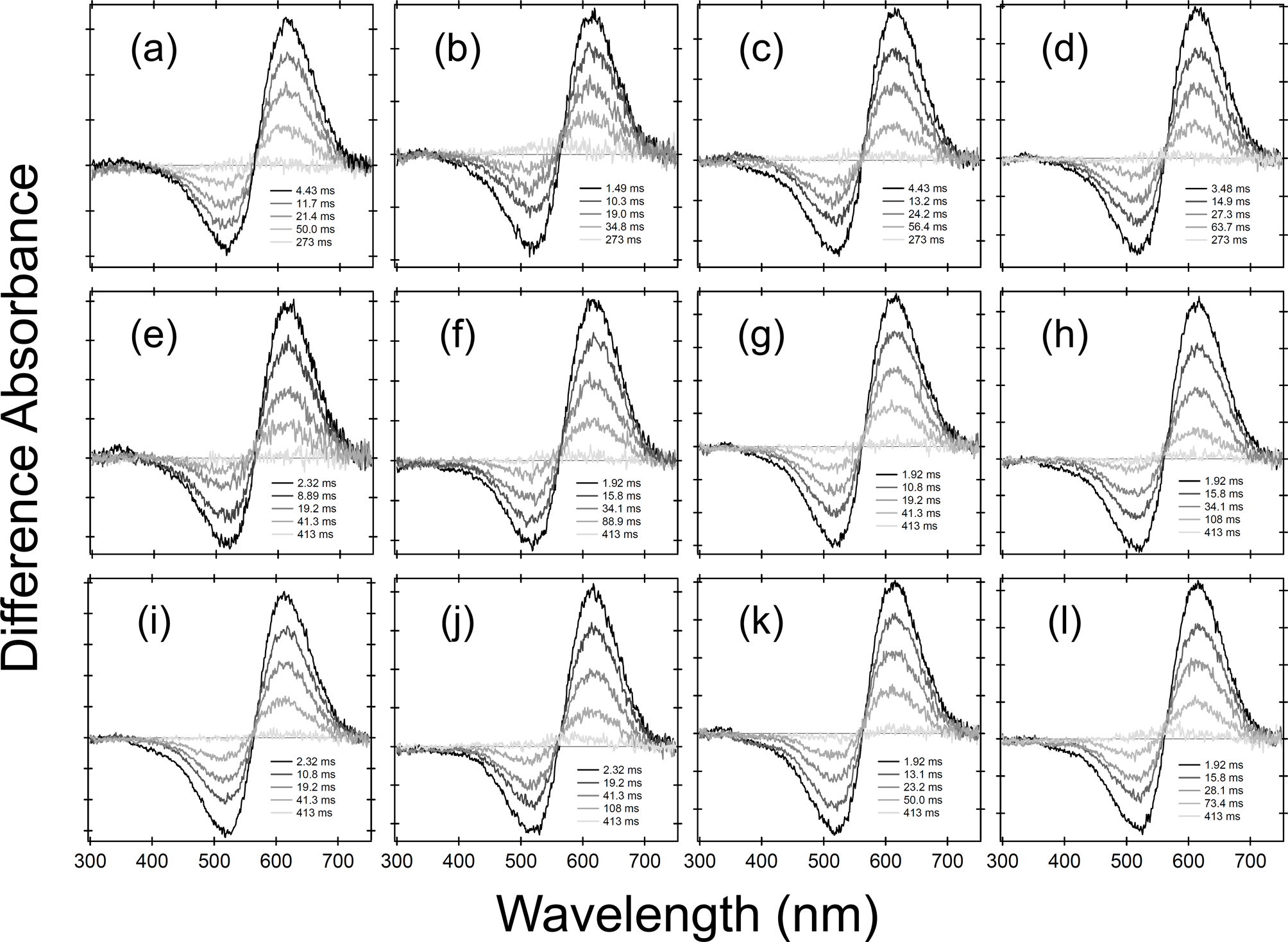

Supplement: Figure S5 — Transient absorption spectra of GR/Rh chimeras. GR/Rh223-252 (a), GR/Rh223-253 (b), GR/Rh225-251 (c), GR/Rh225-252 (d), GR/Rh226-244 (e), GR/Rh227-244 (f), GR/Rh228-244 (g), GR/Rh229-244 (g), GR/Rh226-247 (i), GR/Rh227-247 (j), GR/Rh228-247 (k), and GR/Rh229-247 (l). One division of the y-axis corresponds to 0.01 absorbance units. All samples were solubilized in 0.1% DDM solution. (TIF) [file pone.0091323.s005.tif]

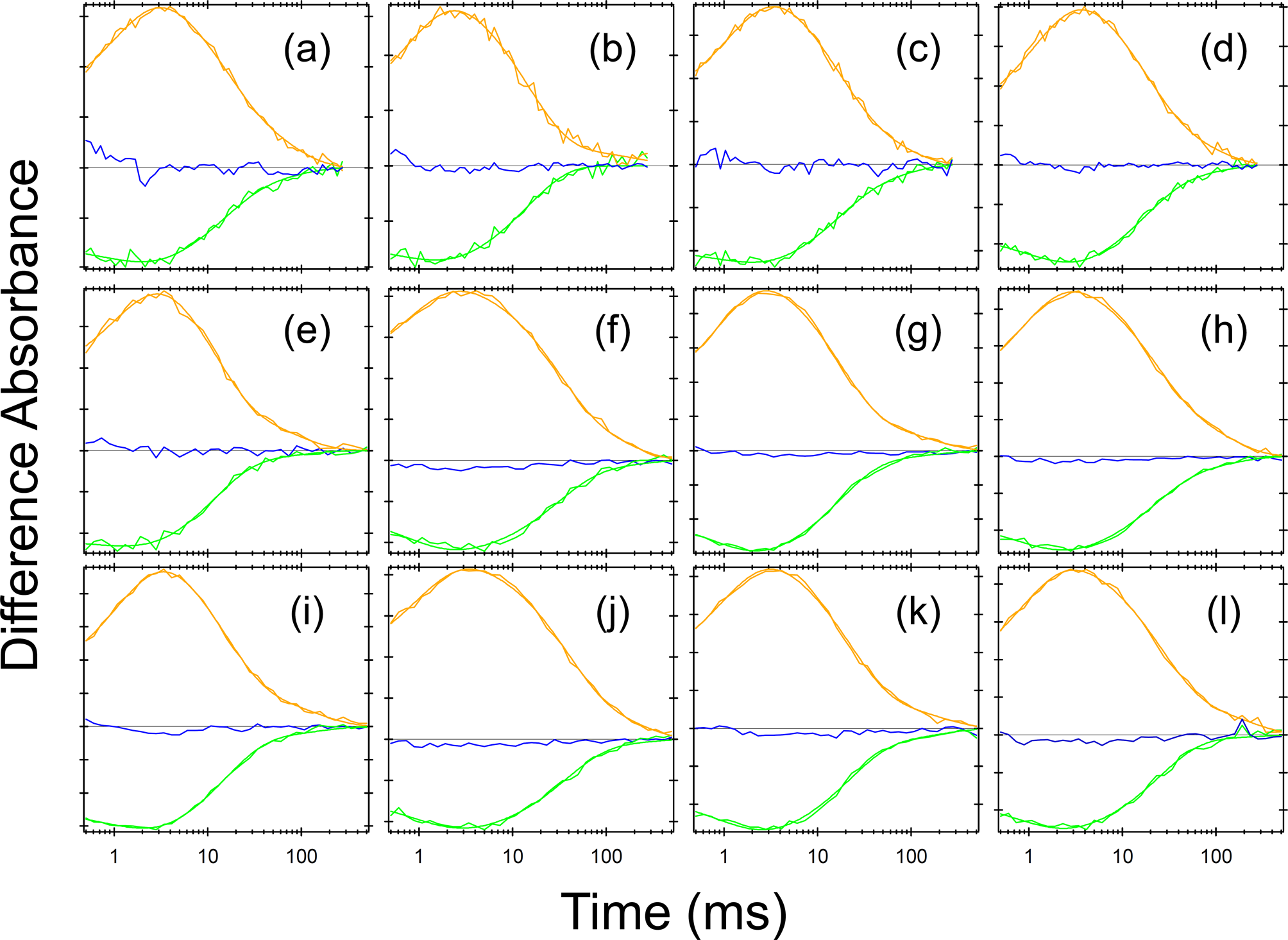

Supplement: Figure S6 — Laser flash photolysis results of GR/Rh chimeras at 25°C. Light-induced absorbance changes of GR/Rh223-252 (a), GR/Rh223-253 (b), GR/Rh225-251 (c), GR/Rh225-252 (d), GR/Rh226-244 (e), GR/Rh227-244 (f), GR/Rh228-244 (g), GR/Rh229-244 (g), GR/Rh226-247 (i), GR/Rh227-247 (j), GR/Rh228-247 (k), and GR/Rh229-247 (l) monitored at 400 nm (blue), 520 nm (green), and 615 nm (orange), which indicate the M intermediate accumulation, the depletion of GR, and the accumulation of O intermediate, respectively. Solid lines with small noise represent the data points, which were averaged for 50 signals. Smooth solid lines correspond to fitting curves (green; triple exponential, orange; triple exponential of rise and two components of O decay). All divisions in the y-axis correspond to 0.01 absorbance units. (TIF) [file pone.0091323.s006.tif]

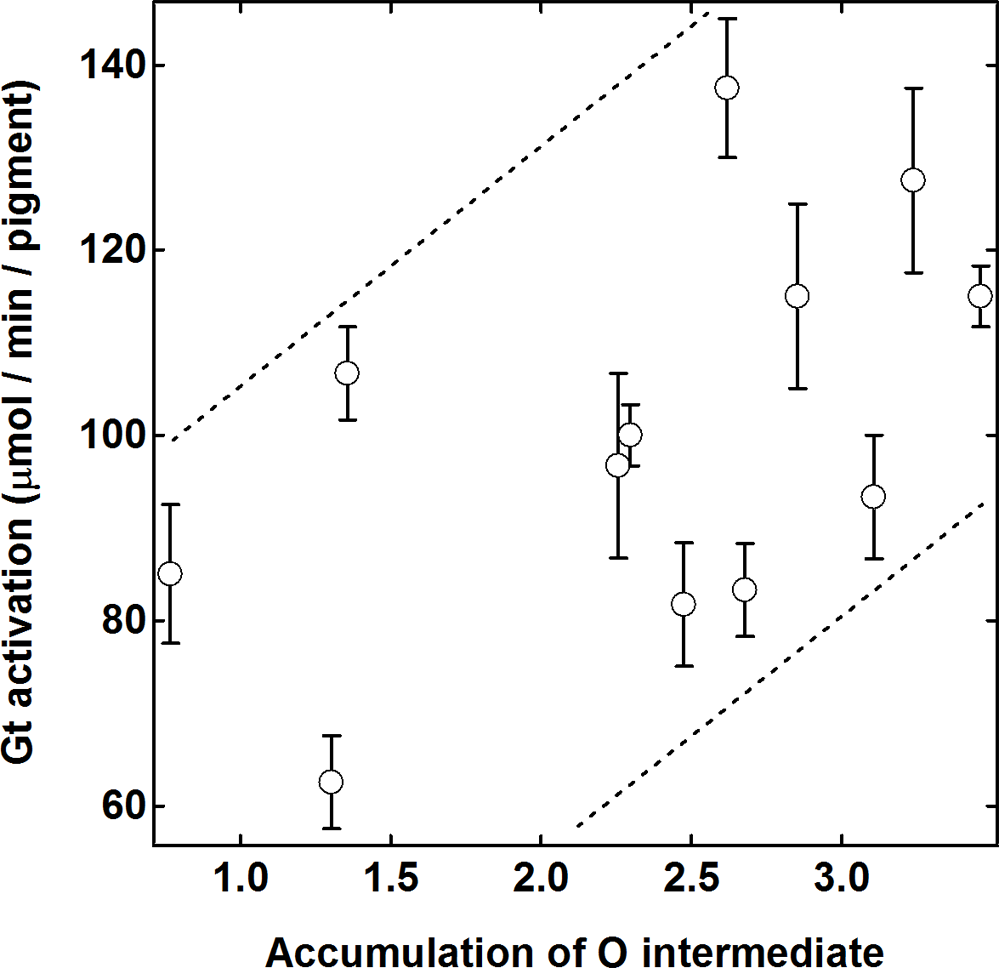

Supplement: Figure S7 — Correlation between the time constant of the decay of O intermediate of bovine Rh third loop inserted GR chimera and the G-protein activation ability. The time constant of the O intermediate decay was calculated in Table 2. The value of G-protein activation ability was calculated using Figure 3c by subtracting [35S]GTPγS bound in dark condition from GTPγS bound in light condition as shown in Table 1. Data are presented as the means ± S.D. of more than three independent experiments. (TIF) [file pone.0091323.s007.tif]

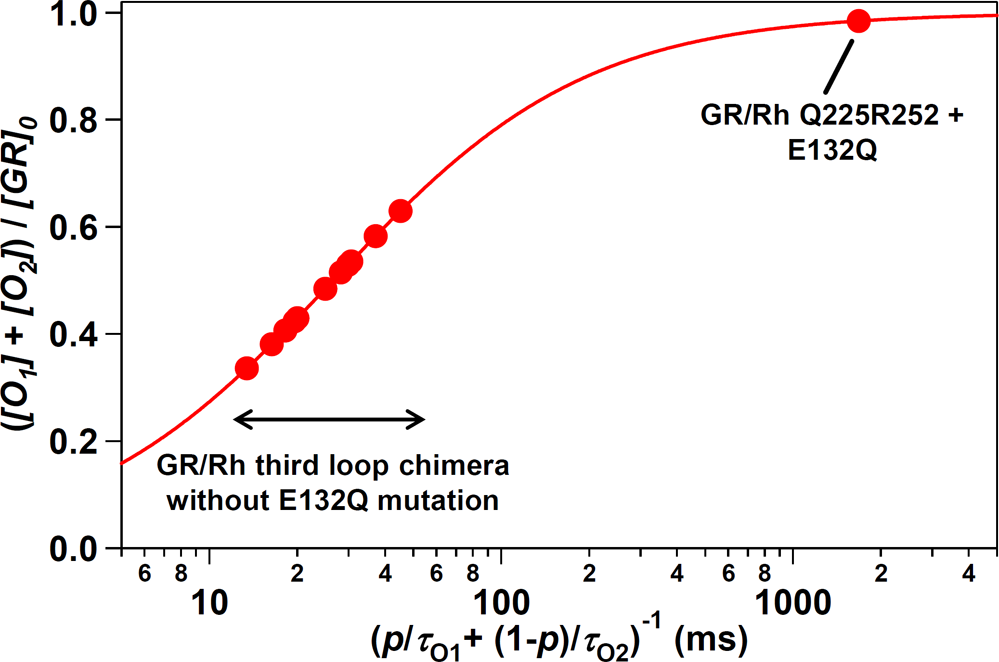

Supplement: Figure S8 — The estimated correlation between the amounts of the accumulated O intermediate in G-protein activation assay with the decay rate of the intermediate (solid line). Red circles represent the values for the mutants constructed in this study. The way of calculation is described in Document S1. (TIF) [file pone.0091323.s008.tif]
