# Supplementary material for: Chimeric Proton-Pumping Rhodopsins Containing the Cytoplasmic Loop of Bovine Rhodopsin
Source: PLoS One. 2014 Mar 12;9(3):e91323. doi: 10.1371/journal.pone.0091323 (PMC3951393; doi:10.1371/journal.pone.0091323)
Supplement: Document S1 — The way of the calculation of the estimation of the amounts of the accumulated O intermediate in G-protein activation assay is explained. (DOC) [file pone.0091323.s009.doc]

**Supplementary Information**

***Estimation of the accumulation of the O intermediate on the G-protein activation assay***

The amount of the accumulation of the O intermediate on the G-protein activation assay can be estimated by solving the rate equation including O-generation by light absorption by rhodopsin and the recovery from O to dark state. In this case, the equation is

$\frac{d\left[O_{1}\right]+\left[O_{2}\right]}{dt}=-\frac{p}{\tau_{O1}}\left[O_{1}\right]-\frac{\left(1-p\right)}{\tau_{O2}}\left[O_{2}\right]+\frac{I_{photon,abs}}{N_{A}V}\Phi$ (1)

where [*O*_1_], [*O*_2_], *p*, *τ*_O1_, *τ*_O2_, *I*_photon, abs_, *N*_A_, *V,* *Φ* are the concentration of O_1_ and O_2_, the fraction of the blanching of reaction to O_1_, the lifetimes of O_1_ and O_2_, the number of photon absorbed by rhodopsin per second, Avogadro number, volume of the sample solution and quantum yield of the photo reaction, respectively. *I*_photon, abs_ can be calculated by the incident photon number per second (*I*_0_) Lambert-Beer relation law as

$I_{photon,abs}=\int I_{photon,0}\left(\lambda\right)\left(1-10^{-\varepsilon\left(\lambda\right)l\left[GR\right]}\right)d\lambda$ (2)

where *ε*(*λ*), *l* and [*GR*] are the molecular extinction coefficient of GR at wavelength *λ*, optical path length and the concentration of GR, respectively. At photo-equilibrated condition, d[*O*_1_]+ [*O*_2_]/dt in Eq. (1) becomes zero and we can calculate the proportion of O to the total concentration [GR]_total_ ([GR]_total_ = [*O*_1_]+ [*O*_2_]+ [*GR*]) as

$\frac{\left[O_{1}\right]+\left[O_{2}\right]}{\left[GR\right]_{total}}=\frac{\ln10\frac{\Phi l}{N_{A}V}\int I_{0}\left(\lambda\right)\varepsilon\left(\lambda\right)d\lambda}{\ln10\frac{\Phi l}{N_{A}V}\int I_{0}\left(\lambda\right)\varepsilon\left(\lambda\right)d\lambda+\frac{p}{\tau_{O1}}+\frac{\left(1-p\right)}{\tau_{O2}}}$ (3)

from Eqs (1) and (2). We measured *I*_0_(*λ*) by a calibrated multichannel detector, *ε*(*λ*) was estimated to be 50,000 M^-1^cm^-1^ at absorption maximum , and *p*, *τ*_O1_ and *τ*_O2_ were determined as Table 2. In addition, *l* = 4.5 mm and *V* = 20 μl in our experimental condition. Based on these information, ([*O*_1_]+[ *O*_2_])/[*GR*] can be calculated as Figure S8 for each mutant.

**References**

57. Imasheva ES, Balashov SP, Choi AR, Jung KH, Lanyi JK (2009) Reconstitution of Gloeobacter violaceus rhodopsin with a light-harvesting carotenoid antenna. Biochemistry 48: 10948-10955.
